# Structure of Ca^2+^-binding protein-6 from *Entamoeba histolytica* and its involvement in trophozoite proliferation regulation

**DOI:** 10.1371/journal.ppat.1006332

**Published:** 2017-05-15

**Authors:** Deepshikha Verma, Aruna Murmu, Samudrala Gourinath, Alok Bhattacharya, Kandala V. R. Chary

**Affiliations:** 1 Department of Chemical Sciences, Tata Institute of Fundamental Research, Mumbai, India; 2 Tata Institute of Fundamental Research, Center for Interdisciplinary Sciences, Hyderabad, India; 3 School of Life Sciences, Jawaharlal Nehru University, New Delhi, India; University of Virginia Health System, UNITED STATES

## Abstract

Cell cycle of *Entamoeba histolytica*, the etiological agent of amoebiasis, follows a novel pathway, which includes nuclear division without the nuclear membrane disassembly. We report a nuclear localized Ca^2+^-binding protein from *E*. *histolytica* (abbreviated hereafter as EhCaBP6), which is associated with microtubules. We determined the 3D solution NMR structure of EhCaBP6, and identified one unusual, one canonical and two non-canonical cryptic EF-hand motifs. The cryptic EF-II and EF-IV pair with the Ca^2+^-binding EF-I and EF-III, respectively, to form a two-domain structure similar to Calmodulin and Centrin proteins. Downregulation of EhCaBP6 affects cell proliferation by causing delays in transition from G1 to S phase, and inhibition of DNA synthesis and cytokinesis. We also demonstrate that EhCaBP6 modulates microtubule dynamics by increasing the rate of tubulin polymerization. Our results, including structural inferences, suggest that EhCaBP6 is an unusual CaBP involved in regulating cell proliferation in *E*. *histolytica* similar to nuclear Calmodulin.

## Author summary

*E*. *histolytica*, the etiological agent of amoebiasis, is a protozoan parasite responsible for around 100,000 deaths per year in developing nations. Though the organism has been identified more than 100 years back, there is not much understanding about the biology of this organism. Calcium signaling plays an important role in the biology of this organism. Here we show structure-functional relationship of one of the Ca^2+^-binding proteins (abbreviated as EhCaBP6) and suggest its involvement in cell division in this parasite. EhCaBP6, a nucleo-cytosolic Ca^2+^-binding protein, is a microtubule end binding protein and overexpression of its gene induces an increase in number of microtubular assemblies in *E*. *histolytica*. Cell division cycle in *E*. *histolytica* occurs along the microtubular structures without disruption of nuclear envelope. Occurrence of multinucleated cells in culture suggests duplication and reduplication of nuclear DNA without cytokinesis. Although Kinesin like protein (Klp1), Formin1 and EhCaBP6 were shown to be part of the microtubular assembly, their role in regulation of the cell cycle is not yet documented. Further, *E*. *histolytica* does not have a typical CaM like protein. However, the 3D structure of EhCaBP6 with two Ca^2+^-binding sites is similar to CaM, in spite of their low sequence similarity. Here, we demonstrate that EhCaBP6 regulates cell cycle specifically by facilitating DNA synthesis, transition from G1 to S phase and cytokinesis. The structural and functional similarity between EhCaBP6 and CaM suggests EhCaBP6 to be a functional homologue of nuclear CaM with important roles in regulation of cell cycle.

## Introduction

Ca^2+^ and Ca^2+^-binding proteins (CaBPs) such as Calmodulin (CaM), Centrins and Annexins have been implicated in cell cycle regulation and progression in many eukaryotes [1,2]. The *in-vivo* and *in-vitro* cell culture studies have revealed spikes in the levels of Ca^2+^ at the G1 and G1/S boundaries [3,4]. The nuclear localized CaM plays the role of a major signal-transducing factor during the cell cycle [2,5]. CaM associates with the microtubule organizing centre (MTOC) and acts as the key molecule that couples cell cycle with Ca^2+^ signaling [6,7]. The levels of intracellular CaM were found to increase during the G1/S transition in normal human fibroblasts [8]. Centrins, also known as Caltractins, are a family of EF-hand Ca^2+^-binding phosphoproteins found in the centrosomes of eukaryotes. Centrins are present in the centrioles and they are required for the centriole duplication [9,10]. They are also considered to play a role in severing of microtubules by causing calcium-mediated contraction. Similar function has been established for the Centrin homologue in yeast, CDC31 [11]. Cells in the mutant strain of *Saccharomyces cerevisiae*, lacking this protein, do not divide despite having grown abnormally large and having accumulated double the amount of DNA. Centrins undergo phosphorylation during G2/M phase boundary, thus assisting in the chromosome separation and mitotic spindle assembly [12]. Moreover, CaBP Annexin II was shown to have an increased expression in HeLa cells at the G1/S and S/G2 boundaries [13]. Taken together, a significant role of Ca^2+^ and CaBPs emerges in the regulation of cell cycle progression.

*Entamoeba histolytica* encodes twenty-seven Ca^2+^-binding proteins (CaBPs): a fact alluding to the existence of an extensive Ca^2+^-signaling system in this protist [14,15]. The structural and functional characterization of a few of these EhCaBPs has implicated their role in phagocytosis and pathogenesis [16–18]. However, as observed in other organisms many of these CaBPs have the potential to be involved in a diverse array of cellular functions. Interestingly, the homolog of the ubiquitous Ca^2+^-binding protein CaM present in almost all eukaryotic cells, has not been found in *E*. *histolytica*, though there is a database entry in the name of CaM. Sequence analysis revealed EhCaBP3 as the closest homolog (49% sequence identity) among all CaBPs. However, functionally EhCaBP3 is found to be distinctly different from CaM [17]. This is also reflected in sequence similarity analysis as CaMs of different species are highly conserved. One of these CaBPs, designated as EhCaBP6, has been reported to be abundantly localized in the nucleus near the microtubule ends and also concentrated along the intercellular bridge with the microtubules during the cell division [19]. The overexpression of EhCaBP6 was correlated with a significant increase in the number of microtubular assemblies suggesting that this protein is involved in the regulation of chromosomal segregation and cytokinesis in this protozoan [19]. Cell division cycle in *E*. *histolytica* has been shown to occur along the microtubular assemblies without disruption of the nuclear envelope [20,21]. Occurrence of multinucleated cells in cell culture suggested duplication and reduplication of nuclear DNA without cytokinesis [22,23]. Although Kinesin like protein (Klp1), Formin1 and EhCaBP6 were shown to be part of the microtubular assembly [19,24,25], their role in regulation of the cell cycle is not yet documented. Further, although CaM activity was biochemically shown in *E*. *histolytica*, the presence of a typical CaM protein with high sequence and functional similarity is not yet reported [26].

**Table 1:** 

EF-I	DRDYDGKIDVKQ
EF-II	FAIEGETFQIEQ
EF-III	DQDKDGKIKASD
EF-IV	GITMESDIDLAT
Scheme I	

We set out to determine the 3D solution NMR structure of EhCaBP6 as a prelude to understand the structural and functional homologs of EhCaBP6 (if any), and to investigate its role in regulating cell cycle. EhCaBP6 is a monomeric protein with molecular weight of 18 kDa. The 3D structure revealed one unusual (EF-I; defined later), one canonical (EF-III) and two non-canonical (cryptic EF-II and EF-IV) EF-hands (see Scheme I). The cryptic EF-II and EF-IV pair with Ca^2+^-binding EF-I and EF-III, respectively, and thus form a two-domain structure as in the case of CaM and Centrin proteins [27–29]. The observed structural similarity between EhCaBP6 and CaM, despite their low sequence identity, suggests that they may be having similar functions during the cell cycle. Exploring this hypothesis, we investigated the localization and interaction of EhCaBP6 with microtubules at different stages of the cell cycle using confocal imaging, immuno-precipitation and co-sedimentation studies. Further, we investigated the role of EhCaBP6 in the regulation of amoebic cell cycle specifically by facilitating DNA synthesis and transition from G1 to S phase. The downregulation of EhCaBP6 has been shown to affect cellular proliferation (by causing delay in G1-S transition), DNA synthesis and cytokinesis. Overall our results show the importance of EhCaBP6 in the regulation of cell proliferation in *E*. *histolytica*, a feature not observed in other parasites.

## Results

### EhCaBP6 exists as a predominantly α–helical stable monomer

The purity and molecular size of protein was checked using gel-filtration chromatography, mass spectrometry (MALDI-TOF) and dynamic light scattering (DLS). The protein showed a single band in SDS-PAGE (S1A Fig) indicative of its purity and molecular size. The MALDI-TOF data showed a strong peak at 18.488 kDa and a weak peak at 9.240 kDa (S1B Fig), which correspond to mono- and doubly-charged states of the protein, respectively. The hydrodynamic radius as determined by DLS was found to be 2.3 nm, which corresponds to the monomeric form of EhCaBP6 (S1C Fig). These results suggest that the protein exists in a monomeric state under the chosen experimental conditions.

The far-UV circular dichroism (CD) experiments were performed with EhCaBP6 to characterize the secondary structural elements in Ca^2+^-bound form. The far -UV CD spectra exhibited a minima near 208 nm (π–π*) and 222 nm (n-π*), which are characteristic signatures of α–helical conformation [30]. The molar ellipticity in far-UV CD spectra was earlier used to characterize the secondary structure content of the protein [19], which also revealed that protein adopts predominantly an α–helical conformation (S1D Fig).

### Identification of EF-hand motifs and Ca^2+^-binding properties in EhCaBP6

Many Ca^2+^-binding proteins (CaBPs) consist of two domains with a linker having a pair of Ca^2+^-binding motifs in each domain and the metal binding has been shown to be highly cooperative in nature. Study of sequence homology showed the presence of one unusual (EF-I) and one canonical (EF-III) EF-hand in EhCaBP6. The unusual character of the EF-I is attributed to the presence of Gln (Q34) residue at the 12^th^ position of the Ca^2+^-binding loop instead of a highly conserved Glu or Asp. It is worth mentioning here that E to Q point mutation was used in the past to convert an active Ca^2+^-binding site into a cryptic one that does not bind Ca^2+^. Intriguingly, the isothermal titration calorimetry (ITC) data showed that EhCaBP6 binds two Ca^2+^ in a sequential manner, implicating that EF-I retains the ability to bind Ca^2+^ [19]. The binding affinities (K_1_: 1.07×10^5^ ± 1.1×10^4^, K_2_: 4.44×10^3^ ± 1.1×10^2^ M^-1^) were reported to be comparatively lower, which could be attributed to the unusual primary structure of EF-hands. NMR Ca^2+^-titration experiments carried out with apo-EhCaBP6 confirmed the two Ca^2+^-binding sites. The ^15^N-^1^H peaks arising from G28 and G101 at the sixth position of EF-I and EF-III were used as markers to identify the Ca^2+^-binding sites (S2 Fig). The appearance of these two downfield shifted peaks indicated the presence of two Ca^2+^-binding sites. Since NMR can give the amino acid residue level information, we monitored the Ca^2+^-filling pathways and noticed that Ca^2+^ indeed binds sequentially, first to EF-I and then to EF-III (S3 Fig). We have carried out site-directed mutagenesis of residues belonging to EF-I (D23A, D25A) and EF-III (D96A and D98A) in order to confirm their role in binding Ca^2+^. The 2D-[^15^N-^1^H]-HSQC of such mutant recorded in the presence or absence of Ca^2+^ were similar and did not show Ca^2+^-binding signatures (S4 Fig).

### The structure of EhCaBP6

**Fig 1:**
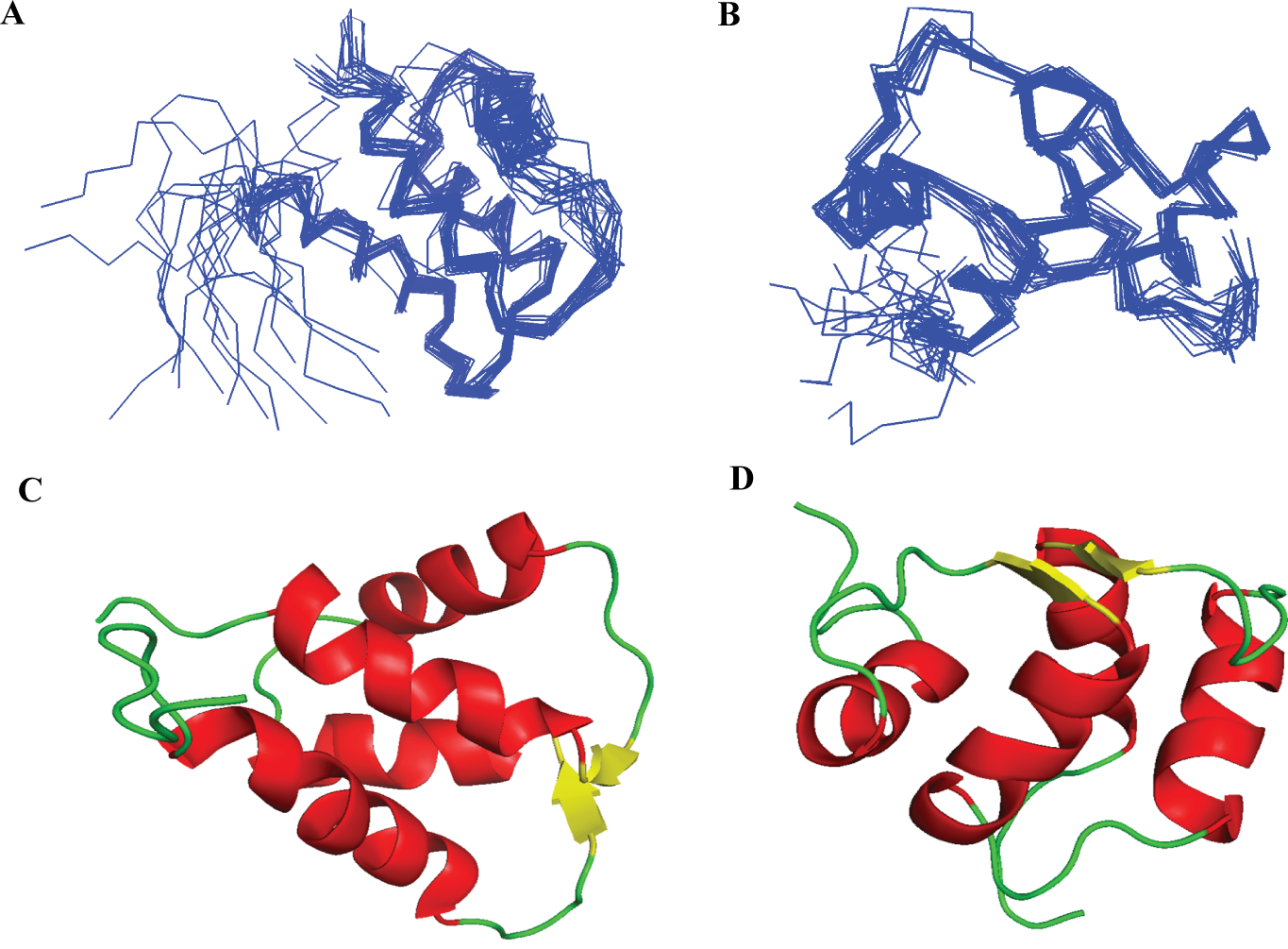
**An ensemble of 20 superimposed minimum energy NMR derived conformers of (A) N-terminal and (B) C-terminal domains of [Ca**^**2+**^**]**_**2**_**-EhCaBP6.** A representative NMR derived solution structure of (C) N-terminal and (D) C-terminal domains of [Ca^2+^]_2_-EhCaBP6 having the least residual target function value among the 20 conformers generated using CYANA.

As mentioned above, EhCaBP6 has one unusual (EF-I), one canonical (EF-III) and two non-canonical (EF-II and EF-IV), EF-hands. NMR and ITC data confirm the EF-I and EF-III to be the two putative Ca^2+^-binding EF-hands. On the other hand, the EF-II and EF-IV were found to be cryptic EF-hands that still pair with the Ca^2+^-binding EF-I and EF-III, respectively, to form a two-domain structure similar to CaM. The 3D structure of EhCaBP6 was determined using NMR data as described in Methods. A superposition of NMR derived 20 minimum energy conformers of the NTD and CTD is depicted in Fig 1A and 1B, respectively. Each domain behaved independently and hence exhibited structural heterogeneity when aligned with respect to one another. NMR structures within a given domain showed good convergence with low root-mean-square-deviations (RMSD) of 0.7 and 0.6 for NTD and CTD, respectively and well-defined tertiary folds. The structural statistics shown in Table 2 support this conclusion. The minimum energy structures of EhCaBP6, having the least residual target functions were chosen as representative structures of NTD and CTD as shown in Fig 1C and 1D, respectively. The central linker region however exhibits large flexibility, suggesting the spatial heterogeneity of the two-domains, with an overall RMSD of 7.0 for full-length protein (S5 Fig; *pdb id*: 5B7X). Poor convergence of the full-length protein was due to the lack of sufficient nOes in the linker region and also due to the absence of any inter-domain long-distance constraints. As mentioned earlier, the EF-I exhibits an unusual EF-hand with a Gln residue at its 12^th^ position of the Ca^2+^-binding loop. The Ca^2+^-coordination geometry of EF-I of EhCaBP6 was found to be octahedral in geometry (S6 Fig), instead of the pentagonal bipyramidal geometry, which is typically observed in EF-hand CaBPs. The octahedral geometry is similar to that of Mg^2+^-coordination present in other CaM like CaBPs.

**Table 2:** Structural statistics for an ensemble of 20 refined conformers of the N- and C- terminal domains (NTD and CTD) of [Ca^2+^]_2_-EhCaBP6.

	NTD	CTD
**NMR distance and dihedral constraints**		
Distance constraints		
Total NOE	530	473
Intra-residue	250	215
Inter-residue		
Sequential (|*i*–*j*| = 1)	226	202
Medium-range (|*i*–*j*| <4)	30	29
Long-range (|*i*–*j*| > 5)	24	27
Hydrogen bonds	40	38
Total dihedral angle restraints		
ϕ	63	63
ψ	63	63
**Structural statistics**		
Violations (mean and s.d.)		
Distance constraints (Å)	0	0.35±0.12
Dihedral angle constraints (°)	0	0
Max. dihedral angle violation (°)	0	0.47
Max. distance constraints violation (Å)	0	
Deviations from idealized geometry		
Bond lengths (Å)	0.001	0.001
Bond angles (°)	0.2	0.2
Average pairwise r.m.s. deviations** (Å)		
Heavy	1.28	1.16
Backbone	0.72	0.56

** Pairwise r.m.s. deviations were calculated among 20 refined structures.

### EhCaBP6 does not participate in phagocytosis

A number of EhCaBPs (EhCaBP1, EhCaBP3, EhCaBP5) have been shown to participate in phagocytosis, thereby influencing pathogenesis in amoebiasis [16–18]. Each of these proteins performs different roles during phagocytosis. However, unlike EhCaBP1, EhCaBP3 or EhCaBP5, EhCaBP6 was not found at phagocytic cups during the initiation or in phagosomes after completion of phagocytosis (S7 Fig). Taken together, this data suggest that EhCaBP6 does not participate in erythrophagocytosis.

### EhCaBP6 interacts with Ehβ–tubulin in micro-tubular structures

**Fig 2:**
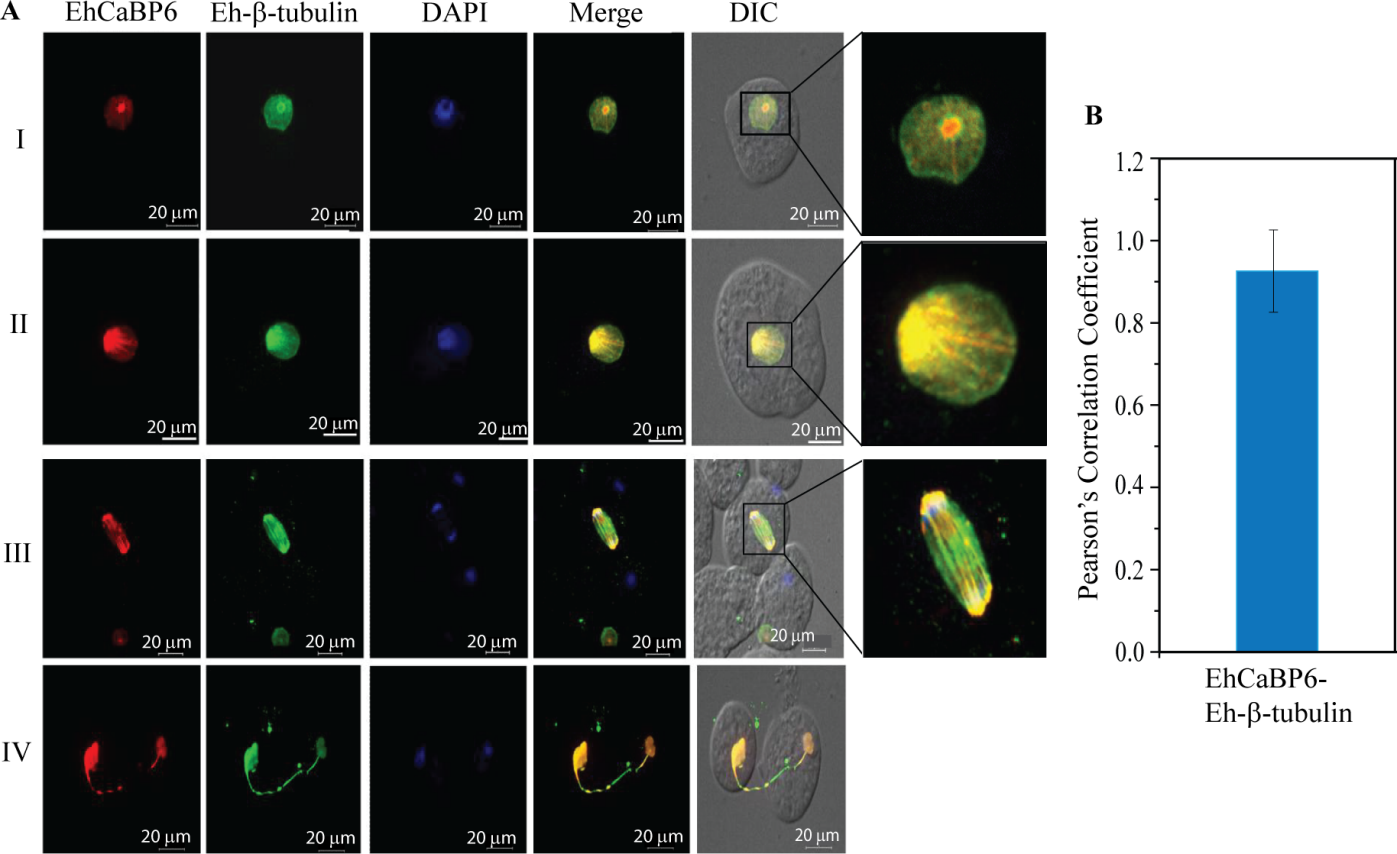
EhCaBP6 co-localizes with microtubules in mitotic structures. (A) EhCaBP6 was observed as intense dot like structure within the nucleus (I), as radial arrays (II), as spindle fibers connecting the dividing nucleus (III) and as an intracellular bridge between the dividing cells (IV). Eh β–tubulin co-localizes with EhCaBP6 in all these structures. EhCaBP6 was stained with Alexa Flour 555 (red) while Ehβ–tubulin was stained with Alexa Flour 488 (green). (B) Statistical analysis of co-localization of EhCaBP6 and Eh β–tubulin using Pearson’s correlation coefficient.

The nuclear EhCaBP6 did not appear to come out of the nucleus at any given instant of time during the cell cycle or during phagocytosis. Its involvement in cell division was proposed to be via association with microtubules [19]. We visualized EhCaBP6 at different stages of the cell cycle using confocal imaging. Cell division in *E*. *histolytica* spans for a period of 8–10 h and during this period, EhCaBP6 was found in various micro-tubular structures that ranged from a speculated *Micro-Tubule Organizing Centre* (MTOC), which was characterized by an intense dot-like structure in the middle of the nucleus, to radial arrays of Ehβ–tubulin radiating from it. EhCaBP6 was thus observed to co-localize with Ehβ–tubulin along the spindle fibers and with the segregated DNA of anaphase-like nucleus. Further, we observed co-localization of EhCaBP6 and Ehβ–tubulin along the intracellular bridge in physically separated cells (Fig 2A). In all of these micro-tubular structures, EhCaBP6 was found to co-localize with Ehβ–tubulin with a Pearson’s correlation coefficient of 0.93 (Fig 2B).

### EhCaBP6 interacts with microtubule and not with monomer of β–tubulin

**Fig 3:**
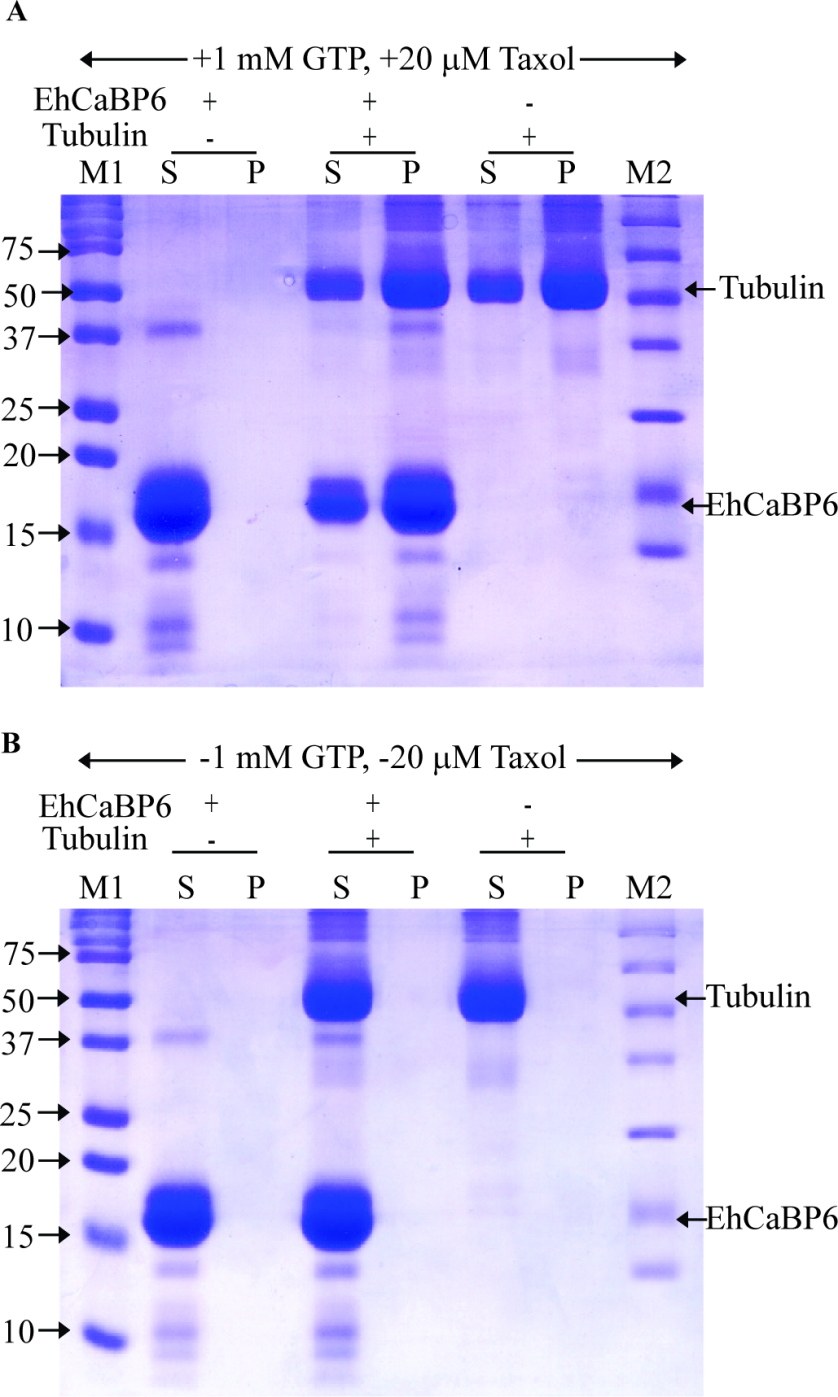
EhCaBP6 interacts with microtubules and not with monomers of β–tubulin. Microtubule co-sedimentation assay was performed using EhCaBP6 and β–tubulin in the presence and the absence of 1 mM GTP and 20 μM Taxol. (A) EhCaBP6 was observed in the pellet fraction along with β–tubulin, thus suggesting *in vitro* interaction between the two proteins. (B) Co-sedimentation assay in the absence of 1 mM GTP shows no interaction.

To validate the interaction between Ehβ–tubulin and EhCaBP6, we carried out EhCaBP6 pull down assay using total HM1 lysate. Ehβ–tubulin was found to be immuno-precipitated by the EhCaBP6 antibody suggesting that these two proteins indeed interact with each other. Intriguingly, we observed that the interaction between EhCaBP6 and Eh β–tubulin did not require Ca^2+^ as the interaction was also noticed in the presence of EGTA (S8 Fig). Further, EhCaBP6 binding to monomeric or polymeric forms of Ehβ–tubulin was tested using *Surface Plasmon Resonance* (SPR) (S9 Fig). Bovine β–tubulin was used in these *in-vitro* experiments due to the availability of highly pure form of the protein. SPR experiment indicated a low binding affinity of 2 mM with the monomeric form of bovine β–tubulin. The interaction between EhCaBP6 and monomeric form of bovine β–tubulin was unaffected by the presence or the absence of Ca^2+^. To test whether EhCaBP6 binds polymerized β–tubulin, microtubule co-sedimentation assay was carried out in the presence and the absence of 1 mM GTP (Fig 3). The results showed an interaction of EhCaBP6 with the microtubule in the presence of 1 mM GTP and 20 μM Taxol. A significant amount of EhCaBP6 was found in the pellet fraction along with microtubules while in the absence of GTP neither EhCaBP6 nor tubulin was present in the pellet fraction. Taken together, we conclude that EhCaBP6 binds microtubule in a GTP dependent manner, while a low affinity binding takes place with the monomeric form of β–tubulin in a GTP independent manner.

### EhCaBP6 modulates microtubule dynamics

**Fig 4:**
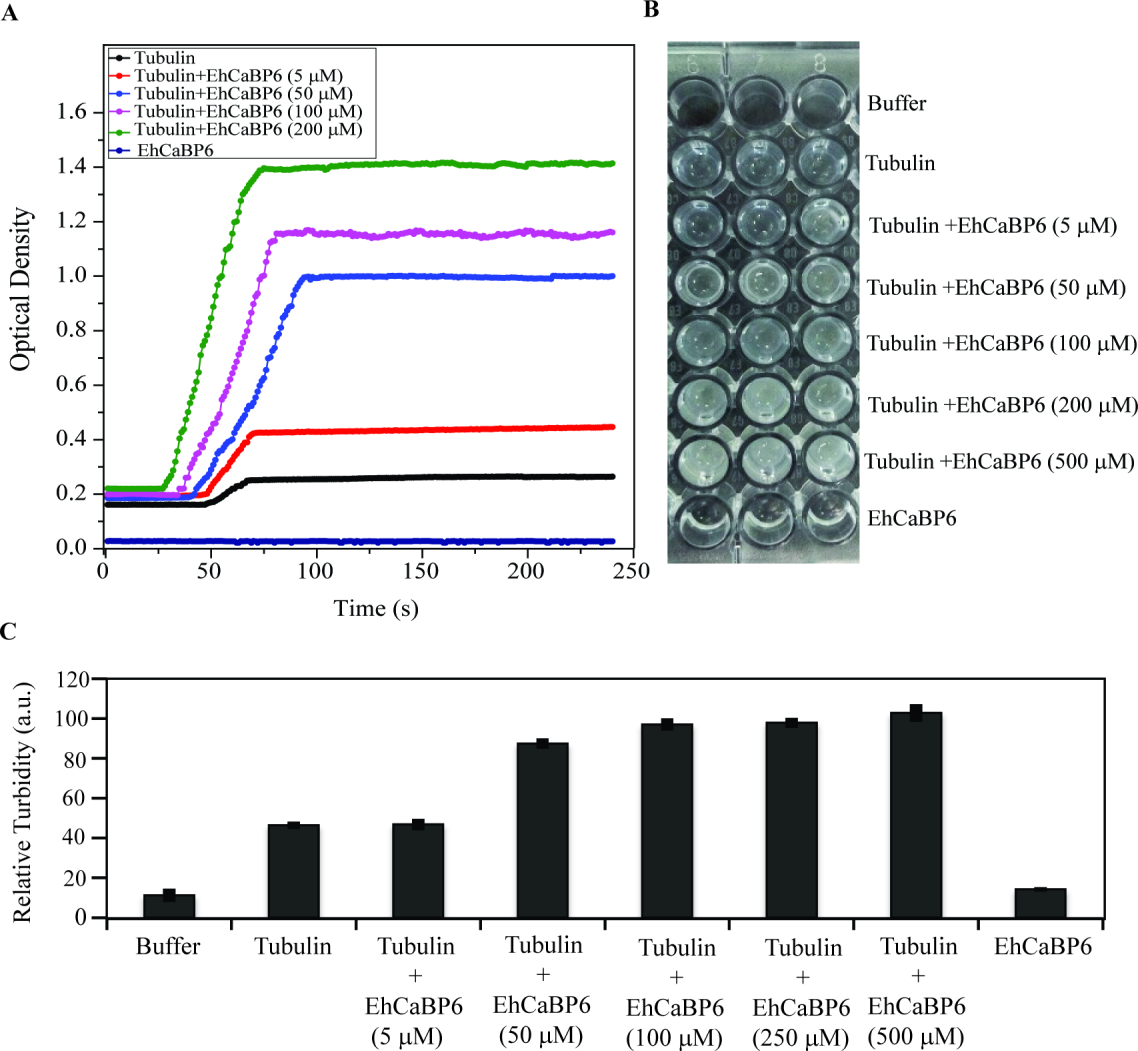
EhCaBP6 modulates microtubule dynamics in a dose dependent manner. (A) The tubulin polymerization assay was performed with porcine tubulin at a final concentration of 5 μM in the presence of varying concentration of EhCaBP6. EhCaBP6 efficiently enhanced rate of polymerization subsequently affecting the total amount of polymerized microtubules. The light scattering experiment was repeated thrice in triplicates and the representation is an average of three independent run. (B) Image of the plate post tubulin polymerization light scattering experiment showing gradual increase in turbidity with increase in EhCaBP6 concentration as compared to Buffer alone, EhCaBP6 alone or Tubulin alone. (C) Densitometry analysis of the turbidity using AlphaEaseFc software. The reading was exported and plot in Microsoft Excel. The average value of three independent experiments has been represented graphically.

Our analysis suggested that EhCaBP6 associates with microtubules. We employed light scattering assay to investigate the role of EhCaBP6 in modulating microtubule dynamics by studying its effect on tubulin polymerization. In the presence of EhCaBP6, we observed enhanced rates of tubulin polymerization in a dose dependent manner (Fig 4). The rate of tubulin polymerization in the presence of EhCaBP6 was twofold as compared to its absence. Besides, EhCaBP6 alone did not show self-polymerization, thus suggesting that EhCaBP6 indeed affects microtubule dynamics. Further, densitometric analysis of the observed turbidity in each reaction (post assay) showed increased turbidity with an increase in EhCaBP6 concentration (Fig 4B and 4C).

### EhCaBP6 downregulation affects cell proliferation and DNA synthesis

**Fig 5:**
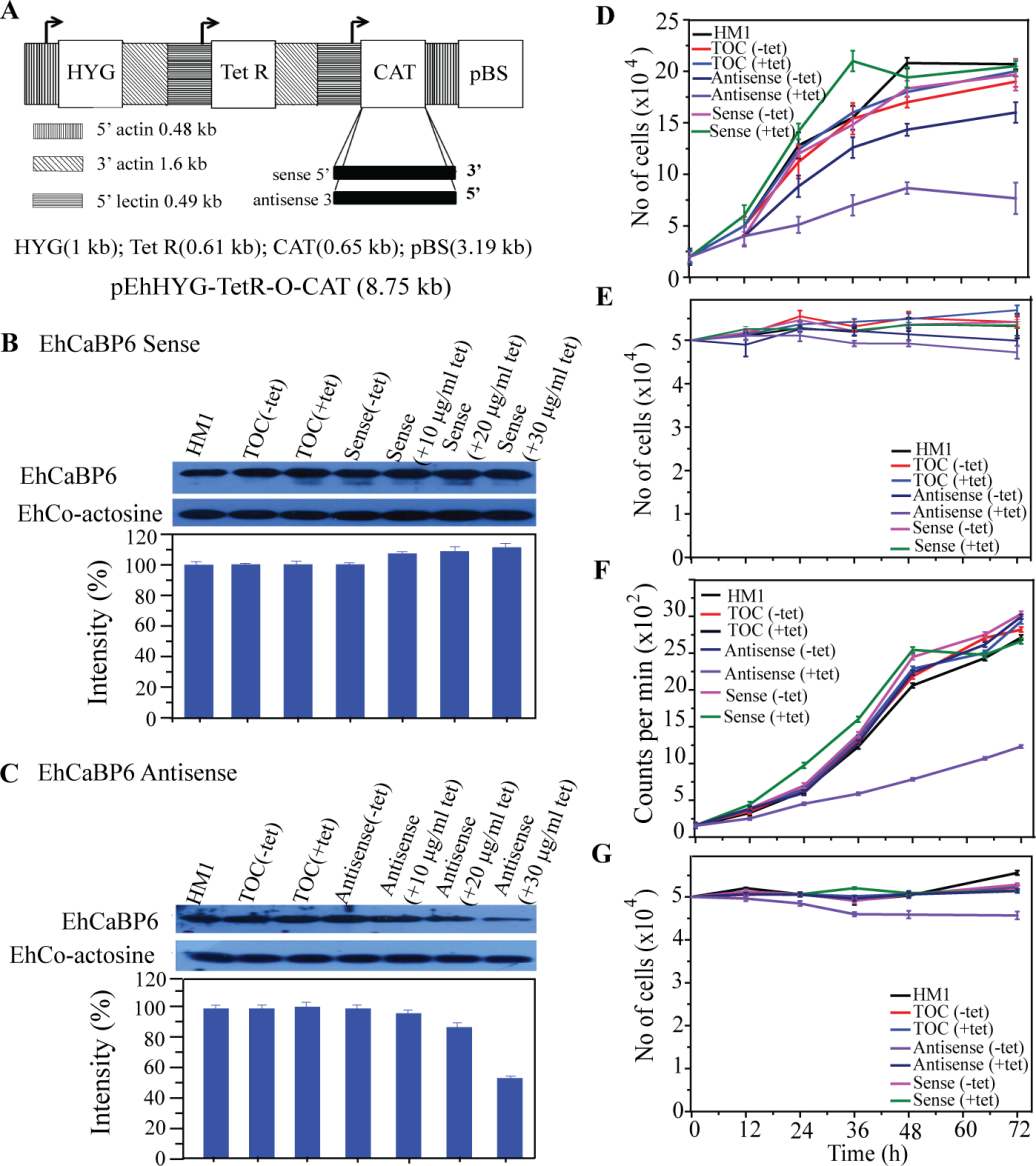
EhCaBP6 downregulation affects cell proliferation and DNA synthesis. (A) Schematic representation of Tetracycline induced Tet-O-CAT vector used for generation of sense and antisense cell lines of EhCaBP6. (B) Analysis of *in-vivo* expression of EhCaBP6 in sense cell line upon induction with varying concentration of tetracycline by Western blot assay. The lower panel represents densitometry analysis of the protein bands obtained from Western blots. (C) Analysis of *in-vivo* expression of EhCaBP6 in anti-sense cell lines upon varying concentration of tetracycline and densitometric analysis of bands from western blots using AlphaEaseFC software. (D) Growth kinetics of various cell lines over a period of 72 h. Equal number of cells were inoculated and allowed to grow. The cells were harvested at indicated time points, resuspended in PBS and counted in a cell counter. (E) Cell viability during the growth kinetics assay after mixing with 0.4% Trypan blue. (F) H3 –Thymidine incorporation assay and (G) Cell viability assay studied along a period of 72 h using Trypan blue.

The role of EhCaBP6 in cell proliferation was further studied by expression downregulation using the gene in sense and antisense orientation in tetracycline inducible vector Tet-O-CAT (Fig 5A). Cell lines harboring the constructs of EhCaBP6 were analyzed for expression of the gene post-induction with varying concentration of tetracycline. The western analysis of total lysate probed with anti-m-EhCaBP6 antibody revealed significant reduction (50%) in expression of EhCaBP6 in antisense cell line upon induction with 30 μg/ml of tetracycline as compared to wild-type HM1, tetracycline induced vector control (TOC) or uninduced antisense cell lines (Fig 5C). However, the sense cell line of EhCaBP6 did not show significant elevation in expression of EhCaBP6 even at 30 μg/ml tetracycline induction (Fig 5B). EhCaBP6 being a nucleo-cytosolic protein, the effect of its downregulation was studied on the rate of cell proliferation and DNA synthesis. Cell proliferation monitored over a period of 72 h suggested significant (p≤0.001) reduction in antisense cell lines without affecting the viability of cells as determined by trypan blue staining (Fig 5D and 5E). H^3^-Thymidine assay exhibited significantly (p≤0.001) reduced rate of DNA synthesis in antisense cell line as compared to normal HM1 cells, vector control (TOC) and sense cell line of EhCaBP6 (Fig 5F). Cell viability was not affected during the course of the study (Fig 5G). This suggests that EhCaBP6 has a role in DNA synthesis and cell proliferation.

### EhCaBP6 downregulation perturbs and delays transition from G1 to S phase

**Fig 6:**
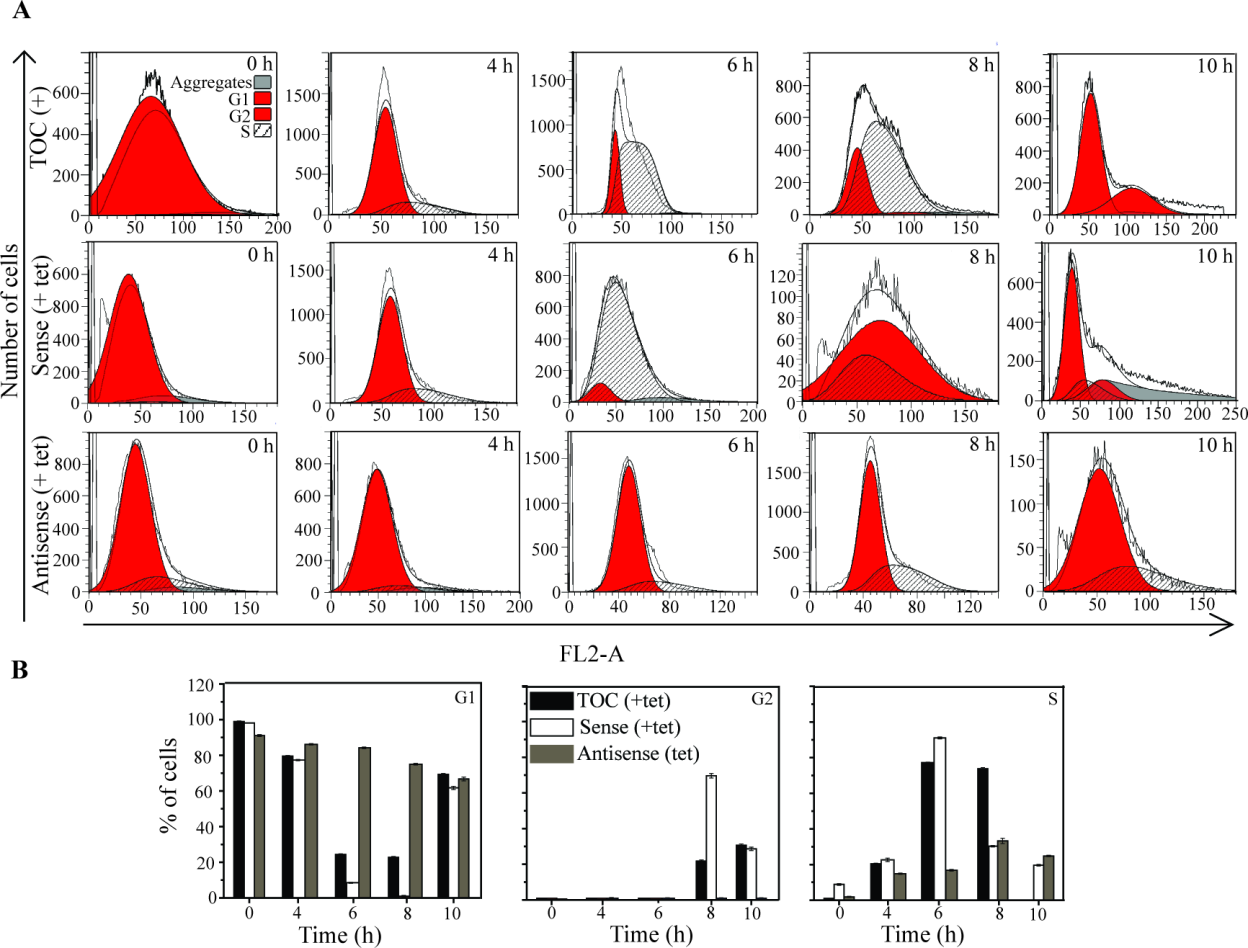
EhCaBP6 perturbs G1 to S phase transition during cell division cycle in *E*. *histolytica*. (A) FACS analysis of cell population during cell division cycle in tetracycline induced vector control (TOC), Sense EhCaBP6 and Antisense EhCaBP6 cell line. (B) Histograms depicting cell populations obtained using MODFIT software. Each of these experiments was performed thrice and each plot is an average of three independent data sets.

H^3^-thymidine incorporation assay suggested that EhCaBP6 affects DNA synthesis. FACS analysis of G1 synchronized cell culture was performed to clearly demarcate which phase of the cell division cycle was inhibited. In tetracycline induced vector control (TOC), 99% of cells were obtained in G1 phase at 0 time. The results are shown in Fig 6A. At 4 h, post-induction mixed population of cells were obtained containing 79% cells in G1 phase and 21% cells in S phase whereas no G2 phase was observed. On further incubation proportion of cells in G1 further decreased with concomitant increase of cells at S phase. At 8h, G2 phase cells start appearing and by 10 h no S phase cells were seen suggesting that the cells were undergoing next round of cell division and DNA synthesis occurs between 4 to 8 h of cell division cycle in normal cells. Based on these results, we infer that in normal proliferating cells, (Fig 6A), DNA synthesis occurs between 4–8 h of cell division cycle.

In the sense cell line of EhCaBP6, significant percent of cells (91%) were observed in S phase at 6 h, subsequently the number gradually declined. At 8 h, we visualized G2 cell population (69%), with 31% cells still in S phase and no G1 phase cell population. However, at 10 h we could see reappearance of G1 cell population (61%) suggesting that the cells are undergoing next round of cell division. Contrary to the vector control and sense cell lines, the antisense cell line failed to transit from G1 phase with a small fraction of cells (25%) in S phase and no G2 phase cells till 10 h. Thus, we conclude that EhCaBP6 down-regulation perturbs DNA synthesis (S phase) besides the transition from G1 to G2 phase. This is likely to be a possible reason behind reduced cell proliferation. Quantitative analysis of cells at different phases further suggested that there is a delayed S phase in the antisense EhCaBP6 cell line and a failure in transition from G1 to G2 (Fig 6B and S1 Table).

### Eh-β tubulin co-localizes with EhCaBP6 at nuclear periphery in EhCaBP6 antisense cell line

**Fig 7:**
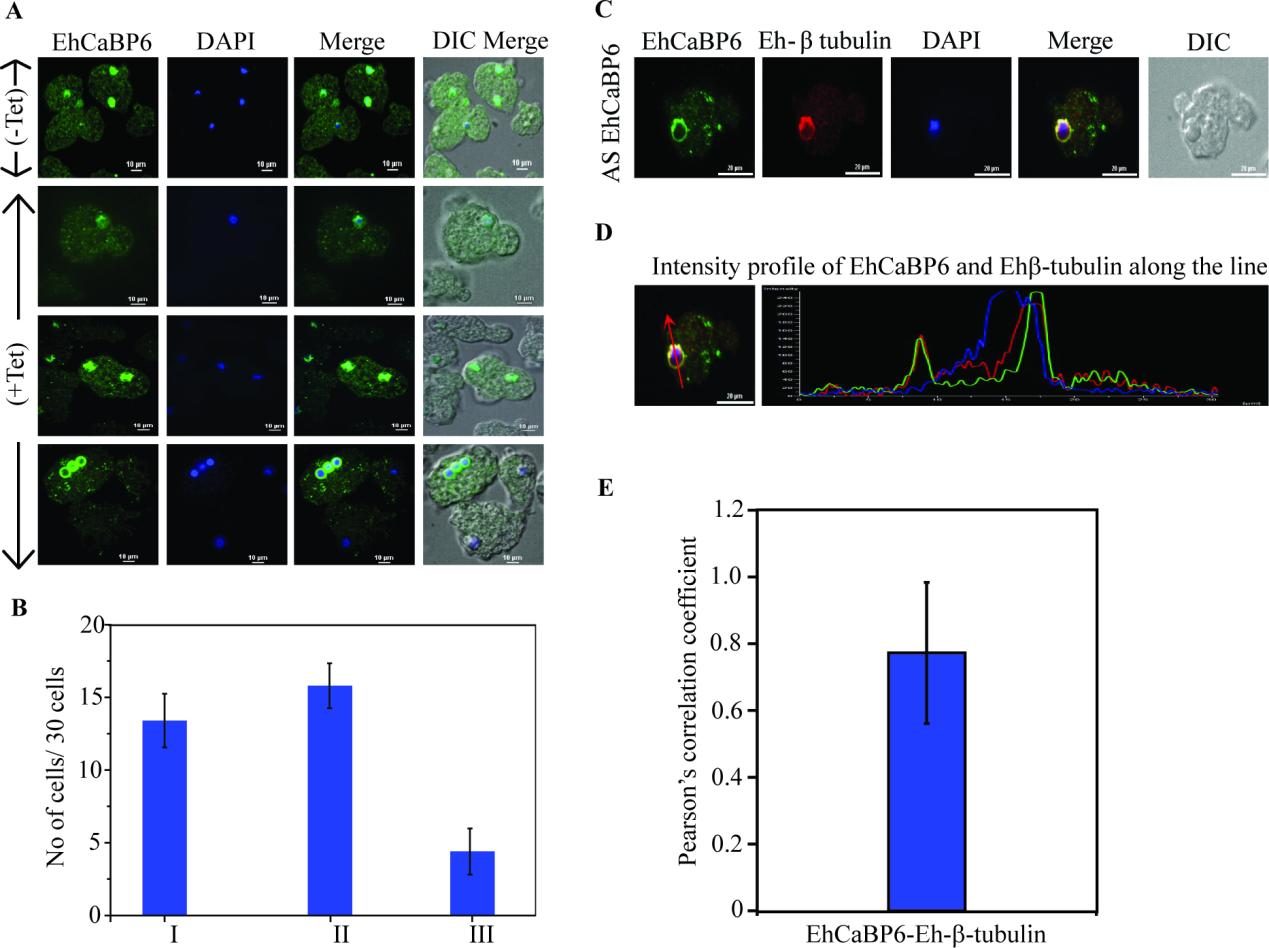
Ehβ-tubulin co-localizes with EhCaBP6 at nuclear periphery in EhCaBP6 antisense cell line. (A) Immunofluorescence analysis of cell population observed in anti-sense EhCaBP6 culture. (B) Quantitative analysis of the various cell populations obtained where (I) Cells having EhCaBP6 in nuclear periphery. (II) Cells with reduced expression of EhCaBP6 and (III) cells with EhCaBP6 expression comparable to normal cells. (C) Immunostaining showing peripheral co-localization of Eh-β tubulin (red) and EhCaBP6 in the nucleus (green) of the EhCaBP6 downregulated cell line. Amoebic cells containing antisense construct were serum synchronized for 24 h followed by serum replenishment and induction with 30 μg/ml of tetracycline. The cells were then fixed and immunostained with specific antibody as indicated. Alexa-488 (green) or Alexa-555 (red) conjugated secondary antibodies were used for visualization. Nucleus was stained with DAPI (blue). (D) Intensity profile of Eh-β tubulin and EhCaBP6 along the nucleus as indicated by red line to visualize co-localization of these molecules in the nuclear periphery. (E) Statistical analysis of co-localization of EhCaBP6 and Eh β-tubulin along nuclear periphery using Pearson’s correlation coefficient.

In the antisense cell line we observed three types of cell populations: (i) cells with expression of EhCaBP6 comparable to that of normal cells, (ii) cells with reduced expression of EhCaBP6, and (iii) cells in which EhCaBP6 lined-up at the periphery of the nucleus (Fig 7A). Since Ehβ-tubulin was observed as a binding partner of EhCaBP6, we attempted to study whether Ehβ-tubulin too lines up at the nuclear periphery with EhCaBP6. Immunostaining studies indeed revealed that Eh-β tubulin also forms a ring like structure along the nuclear periphery, as seen in the case of EhCaBP6 (Fig 7C). Intensity profile along the nuclear periphery further supported the finding that Ehβ-tubulin indeed co-localizes with EhCaBP6 along the nuclear periphery in the down-regulated cell lines (Fig 7D). The two proteins showed a significant co-localization with a Pearson’s correlation coefficient of 0.8 (Fig 7E).

### EhCaBP6 perturbs cytokinesis in *E*. *histolytica*

**Fig 8:**
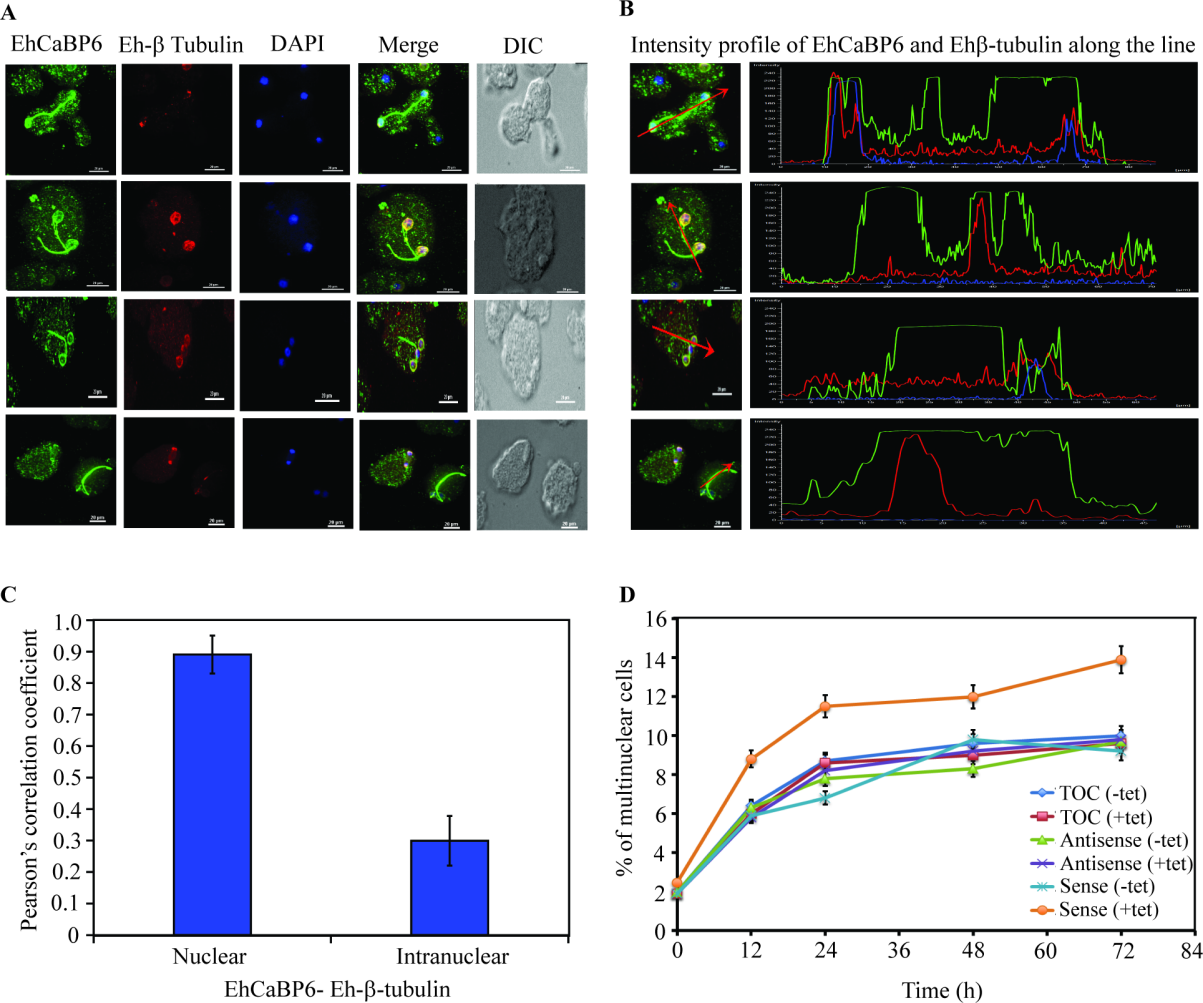
Eh-β tubulin does not co-localize with EhCaBP6 at truncated nuclear structures or intra-nuclear bridge. (A) EhCaBP6 forms truncated intra-nuclear structures within the nucleus in EhCaBP6 downregulated cell line. However, Eh-β tubulin does not line these truncated structures or intra-nuclear bridge as seen from immunostaining data. Amoebic cells containing Antisense construct were serum synchronized for 24 h followed by serum replenishment and induction with 30 μg/ml tetracycline. The cells were then fixed and immunostained with specific antibody as indicated. Alexa-488 (green) or Alexa-555 (red) conjugated secondary antibodies were used for visualization. Nucleus was stained with DAPI (blue). (B) Intensity profile of Eh-β tubulin and EhCaBP6 along the truncated intranuclear structures as indicated by red arrow to visualize co-localization of these molecules. (C) Statistical analysis of colocalization of EhCaBP6 and Ehβ-tubulin along truncated nuclear structures as determined by Pearson’s correlation coefficient. (D) Quantitative analysis of multinucleation in various cell lines under study. The amoebic cells containing the constructs were serum synchronized for 24 h followed by replenishment with complete media and induction with 30 μg/ml tetracycline. The cells were harvested at indicated time points and stained with DAPI to visualize and count the multinucleated cells. The experiment was repeated thrice and 20 fields were analyzed for each experiment.

In normal proliferating cells, EhCaBP6 was observed to line-up the inter-nuclear and inter-cellular bridges along with Eh-β-tubulin. Hence, we attempted to investigate the possible role of EhCaBP6 in cytokinesis. The EhCaBP6 downregulated cell line was serum synchronized and immunostaining was performed after induction with 30 μg/ml of tetracycline. Interestingly, we observed truncated nuclear structures with peripheral nuclear localization of EhCaBP6 (Fig 8A). No significant co-localization of EhCaBP6 and Ehβ-tubulin was observed along the truncated nuclear structures and inter-nuclear bridges as evident from intensity profile analysis and Pearson’s correlation coefficient (Fig 8B and 8C). Thus, suggesting a possible role of EhCaBP6 in cytokinesis. In case of nuclear division followed by failed cytokinesis we expect to observe accumulation of cells with multiple nuclei. Therefore, we quantitated the percentage of multinucleation in various cell lines under study (Fig 8D). Quantitative analysis for a period of 72 h suggested enhanced multinucleation in the antisense cell line as compared to the cells with only the vector or the sense cell line. Taken together we conclude that EhCaBP6 may have a role in cytokinesis.

### Depletion of intracellular Ca^2+^ led to translocation of EhCaBP6 from nucleus to cytoplasm

We hypothesized that Ca^2+^ may play an important role in determining the subcellular localization of EhCaBP6. In order to test this hypothesis, EhCaBP6 localization was studied upon treatment of cells with BAPTA-AM, an intracellular Ca^2+^-chelator (S10 Fig). Immuno-staining was carried out after the treatment of cells with different concentrations of BAPTA-AM for 30 min. The study showed gradual translocation of EhCaBP6 from nucleus to cytoplasm with increasing concentrations of BAPTA-AM. This was validated by cellular fractionation of untreated and 500 μM BAPTA-AM treated HM1 cells followed by western blot analysis of the fractions using anti-m-EhCaBP6 antibody. Immuno-staining of western blotted antigens showed enrichment of EhCaBP6 in the cytoplasmic fraction upon treatment with 500 μM BAPTA-AM. Further, in the presence of ionomycin, that allows increase in intracellular Ca^2+^ level, EhCaBP6 remained inside the nucleus.

Further, we went on to investigate if cytosolic shuttling of EhCaBP6 upon BAPTA-AM treatment is a direct or indirect effect of Ca^2+^ depletion. To begin with we generated a Ca^2+^ defective mutant (EhCaBP6ΔEF) of EhCaBP6 tagged with GFP at the N-terminal and transfected the construct into *E*. *histolytica* trophozoites. Binding studies carried out using ITC of the Ca^2+^ defective mutant confirmed loss of Ca^2+^-binding ability with very low Ca^2+^-binding affinities (S11 Fig). The expression of GFP-tagged EhCaBP6ΔEF and native protein in *E*. *histolytica* trophozoites increased with increasing concentration of G418 as seen in the western blots using anti-GFP antibody (S12A Fig). Imaging of GFP-EhCaBP6ΔEF showed that the Ca^2+^-binding defective mutant also resides in the nucleus similar to that of the native EhCaBP6 (S12B and S12C Fig). Hence, we conclude that the shuttling of EhCaBP6 between cytoplasm and nucleus is indeed an indirect effect of Ca^2+^ depletion and, may be brought about by other cellular or molecular factors.

### Heat stress of EhCaBP6 antisense cell line

We have seen that EhCaBP6 antisense parasites have a defect in cellular proliferation. Upon heat stress, these cells became very fragile and did not attach to the substratum precluding any attempt to carry out expression or phenotype analysis.

## Discussion

Ca^2+^ signaling has been demonstrated as one of the most pivotal pathways in amoebic pathogenesis. However, as pointed out earlier, the influence of Ca^2+^ in amoebic cells has not been extensively investigated till date. The occurrence of twenty-seven distinct CaBPs in *E*. *histolytica* suggests their massive contribution in the regulation of various Ca^2+^-signaling pathways. The involvement of a few CaBPs in phagocytosis and gene expression underlines the need for further detailed studies with other CaBPs [31].

We have determined 3D solution structure of EhCaBP6 by NMR and investigated its role in cell cycle. The results reported here, along with the past reports [19], suggest that EhCaBP6 is functionally different from other EhCaBPs studied so far. Strong evidence of co-localization with microtubules during various stages of the cell cycle and direct interaction with microtubules in a GTP-dependent and Ca^2+^-independent manner illuminated the particulars of its participation in the cell cycle.

The Ca^2+^-signaling inside the nucleus is important in many essential cellular processes including cell division cycle [5]. Ca^2+^ transients are shown to regulate various checkpoints of cell division through CaBPs that activate the downstream processes. This signaling pathway is generally mediated by CaM in other eukaryotic systems [2]. The absence of CaM in the genome of *E*. *histolytica* entails the existence of another functionally analogous candidate involved in cell cycle regulation. EhCaBP6 protein was thus considered as a viable alternative molecule modulating the cell cycle. As nuclear membrane of *E*. *histolytica* does not disintegrate during cell division [21], it is not surprising that EhCaBP6 continues to be localized within the nucleus during the cell division which is important for its involvement in nuclear division. This is in contrast to the behavior of the nuclear EhCaBP3, which migrates to the cytoplasm during phagocytosis [17]. EhCaBP6 can, therefore, be classified into a protein class distinct from EhCaBP3 and other EhCaBPs studied so far.

Our sequence analysis results, complemented with ITC and NMR data, revealed that EhCaBP6 has two Ca^2+^-binding sites (D23-Q34 and D96-D107). The EF-I of EhCaBP6 (D23-Q34) showed unusual Ca^2+^-binding as it has Gln (Q34) at the twelfth position of the Ca^2+^ binding loop instead of the highly conserved Glu/Asp in the other EF hands. The Q34 coordinates to Ca^2+^ through its side-chain amide oxygen atom forming an octahedral geometry (S6 Fig), a feature of Mg^2+^-binding, which was characterized on the basis of the paramagnetic titration experiments, where the side-chain NH_2_ protons of Q34 were seen to completely broaden out in the presence of paramagnetic Ce^3+^. The Ca^2+^-titration data (S2 Fig) of apo- to holo-EhCaBP6 revealed sequential occupancy of the two sites present in EhCaBP6, with the Ca^2+^ binding first to the EF-I in the NTD, followed by its binding to the EF-III in the CTD as monitored by the downfield shift of Gly peaks upon Ca^2+^ binding (S3 Fig).

As reported, the NMR results suggested that the [Ca^2+^]_2_-EhCaBP6 predominantly exists in an α–helical conformation with four short stretches of β-strands [32]. The EF-hands within a protein can have different binding affinities for metal ions, and their pairing results in cooperativity having a functional consequence on Ca^2+^-binding along with maintaining structural stability of the metal binding motif and the domain [33]. Proteins containing four EF-hand motifs usually have two domains. Each of the domains is formed by a pair of EF-hands, usually separated by a flexible linker, which could be extended in forming classical dumbbell structures seen for CaM and troponin C. The structural characterization of EhCaBP1 and EhCaBP2 revealed presence of two-domain structures with four EF-hands [34,35]. On the other hand, EhCaM (EhCaBP3) was shown to possess a pair of EF-hands (EF-I and EF-II) in its NTD and a completely unstructured CTD having a lone unpaired non-canonical EF-hand (EF-III) [36]. The NMR derived 3D-structure of EhCaBP6 showed that EhCaBP6 has one unusual (EF-I; D23-Q34), one canonical (EF-III; D96-D107) and two non-canonical (cryptic) (EF-II and EF-IV) EF-hands. The cryptic EF-II and EF-IV pair with Ca^2+^-binding EF-I and EF-III, respectively, and provide the structural stability to form a two-domain structure similar to CaM and centrin proteins. The structural similarity between EhCaBP6 and CaM, despite their low sequence similarity, provides a compelling evidence for EhCaBP6 as a functional replacement of CaM in cell division. This has further been supported by the increased rate of tubulin polymerization in the presence of EhCaBP6. Localization of EhCaBP6 in the nucleus does not allow this protein to be an overall functional equivalent of CaM. In that sense it displays properties of CaM, associated with cell division.

The downregulation of EhCaBP6 in *E*. *histolytica* cells resulted in decreased cell proliferation and DNA synthesis. We also observed a delayed transition between G1 to S phase and failed cytokinesis upon downregulation of EhCaBP6. Numerous reports on studies of different cell types have proven that Ca^2+^/CaM mediated signaling intricately regulates the cell cycle [1,2]. CaM was reported to be indispensable for DNA synthesis; its inhibition causes delays in G1/S, G2/M and metaphase-anaphase transition. In HeLa cell lines, CaM was reported to be localized with central spindles and abnormal distribution of CaM resulted in inhibition of cytokinesis [6]. Number of studies in mammalian cells have shown that a defect in cytokinesis can lead to polyploidy [37]. In these systems a large number of proteins are involved in not only cytokinesis, but also different check point controls. Unfortunately, very little is known in *E*. *histolytica* except for the presence of a few homologs identified through sequence similarity searches [38]. Therefore, identification of EhCaBP6 as a potential cytokinesis and cell division regulating molecule is likely to help understand this complex process in this important parasite. We conclude from this study that EhCaBP6 is a CaM like protein, which regulates cell cycle in *E*. *histolytica* cells.

## Materials and methods

### Protein expression and purification

The gene encoding EhCaBP6 protein was cloned into a pet21C vector. [Ca^2+^]_2_-EhCaBP6 was overexpressed in *E*. *coli* BL21 (DE3) strain and purified as described earlier [32]. Apo-form of EhCaBP6 was prepared by treatment with EGTA followed by buffer exchange of [Ca^2+^]_2_-EhCaBP6 using a centrifugal ultrafiltration system with a 3 kDa cut-off membrane [39].

### Circular dichroism (CD)

The CD spectra of [Ca^2+^]_2_-EhCaBP6 were recorded on a JASCO J-810 spectropolarimeter equipped with a Peltier temperature controller. Protein concentration of 20 μM was used in recording the far-UV CD spectra for characterizing the secondary structure of the protein at different pH values ranging from 5 to 9.

### Dynamic light scattering

The hydrodynamic radius (R_h_) of EhCaBP6 was determined using dynamic light scattering (DLS) method on a Dynapro-LS instrument at 830 nm. The protein samples were centrifuged at 13,000 rpm for 10 min and filtered through a syringe filter of 0.45 μm into a quartz cuvette. A protein concentration of 8 mg/ml was used in each of the 50 odd measurements. Out of these measurements, only those showing a parabolic curve with a straight base-line were considered for the calculation of the mean values and associated standard-deviation. The R_h_ was then determined from the regularization plot.

### MALDI-TOF

The protein solution (0.5 μl) of 150 μM concentration was mixed with an equal volume of matrix (saturated solution of cyano-4-hydroxycinnamic acid in 50% acetonitrile and 50% TFA (0.1% (v/v) in water). The protein mixture was loaded on to the plate and dried at room temperature. The dried up sample was loaded on to a MALDI-TOF system (BRUKER Ultraflextreme), which is equipped with a smart-beam technology in its linear mode. The data thus collected was analyzed to determine the molecular weight of the protein.

### NMR spectroscopy

NMR experiments were carried out at 25°C with approximately 1 mM protein samples on a Bruker Avance 800 MHz NMR spectrometer equipped with 5 mm triple-resonance cryogenically cooled probe. For NMR studies, unlabeled, uniformly ^15^N-labeled (u-^15^N), fractionally ^13^C-labeled (10%-^13^C) and uniformly ^13^C/^15^N doubly labeled (u-^13^C/^15^N) EhCaBP6 samples were prepared following the protocol described earlier, in a mixed solvent of 90% H_2_O and 10% ^2^H_2_O (50 mM Tris (pH = 6.8), 50 mM NaCl and 4 mM CaCl_2_). Besides, few EhCaBP6 samples were prepared where in certain amino-acid residues were selectively unlabeled, following the procedure described earlier [40]. The unlabeled EhCaBP6 was prepared in both 100% ^2^H_2_O and in an admixture of 90% H_2_O and 10% ^2^H_2_O.

Experiments with u-^15^N or u-^13^C uniformly labeled EhCaBP6 included sensitivity-enhanced 2D [^15^N-^1^H] HSQC and 2D [^13^C-^1^H] HSQC using pulsed-field gradients for coherence selection [41], 3D TOCSY-[^15^N-^1^H] HSQC (τ_m_ = 60 ms) [42], 3D NOESY-[^15^N-^1^H] HSQC (τ_m_ = 80 ms), 3D NOESY-[^13^C-^1^H] HSQC (τ_m_ = 100 ms), 3D HNHA, GFT (3, 2)D HNHA, 3D HNHB, GFT (3, 2)D HNHB and 3D HCCH COSY [43–45]. The experiments recorded with u-^13^C/^15^N doubly labeled samples for backbone and side-chain resonance assignments were as described earlier [32]. The unlabeled EhCaBP6 sample was used to record a 2D [^1^H-^1^H]-NOESY in ^2^H_2_O with a mixing time (τ_m_) of 100 ms. All the spectra were processed using TopSpin 3.1 and analyzed by TATAPRO [46] and Cara [47]. The ^1^H chemical shifts were referenced with respect to an external standard 2,2-dimethyl-2-silapentene-5-sulfonate (DSS), while ^13^C and ^15^N chemical shifts were calibrated indirectly [48,49]. Almost complete ^1^H, ^13^C and ^15^N resonance assignments of EhCaBP6 in its Ca^2+^-bound state (holo-form) were carried out with a suite of double- and triple-resonance NMR experiments as described earlier [50–53] (BMRB accession number 26571) [32].

Paramagnetic titration experiments with [Ca^2+^]_2_-EhCaBP6 were carried out with CeCl_3_ (*Sigma Aldrich*) to monitor Ca^2+^-displacement by Ce^3+^. The protein concentration was 0.5 mM in a buffer containing 50 mM Tris-HCl (pH = 6.8) and 50 mM NaCl. For each titration, an aliquot of 0.5 μL CeCl_3_ of the stock solution (50 mM) was added to the NMR tube containing the protein solution, mixed, equilibrated and followed by recording 2D [^15^N-^1^H] HSQC at 25°C. The HSQC spectra thus recorded were processed with identical processing parameters. Integral volumes for the individual ^15^N-^1^H cross peaks in the resultant spectra were measured using Topspin 3.1.

### NMR structure calculation

The 3D structure of EhCaBP6 was calculated using the information derived from the solution NMR data, which included nOe distance restraints, backbone torsion-angle restraints, restraints due to hydrogen bonds, and Ca^2+^-ligand coordination (described below in the Ca^2+^-Ligand Distance Constraints section) using standard simulated annealing and torsion-angle dynamics protocol with the program Cyana 3.97 [54,55]. The nOe peaks in 3D-[^1^H-^1^H]-NOESY-[^15^N-^1^H]-HSQC,3D-[^1^H-^1^H]-NOESY-[^13^C-^1^H]-HSQC and 2D-[^1^H-^1^H] NOESY spectra were identified, assigned, and linked in the NOESY spectra using the program CARA. A total of 1003 distance constraints, which included 465 intra-residue, 428 inter-residue (sequential), 59 medium-range, and 51 long-range distance constraints, were used in the structure calculation. Hydrogen bond constraints were added for residues that are involved in α–helical and β–sheet conformation. These secondary structural elements were characterized by the chemical shift indices and ^3^J(^1^H^N^-^1^HA) values derived from GFT (3, 2)D HNHA, and were further supported by calculating deuterium exchange rates. A total of 78 H-bond constraints were used during the structure calculation. The dihedral angle constraints were derived from TALOS using the backbone ^1^HA, ^15^N, ^13^CA, ^13^CB, and ^13^CO chemical shift values. A total of 252 (φand ψ) dihedral angle constraints were used in the structure calculation of EhCaBP6 in its Ca^2+^-bound state.

Crystallographic data on several EF-hand proteins suggest that in a canonical Ca^2+^-binding loop there is a strong hydrogen bond formed between the side-chain carboxyl oxygen of Asp at position 1 and the backbone ^1^H^N^ of the Gly residue at position 6. In the present case, the ^1^H^N^ of Gly-28 and Gly-101 are involved in such hydrogen bonding, causing a downfield chemical shift in their spectral signatures. Further, this led to the appearance of corresponding ^15^N-^1^H^N^ correlations 2D [^15^N-^1^H] HSQC in a non-overlapping region. It has also been found from crystallographic studies on several EF-hand CaBPs that Ca^2+^ coordinates directly with the highly homologous residues at positions 1, 3, 5, 7, and 12 of individual Ca^2+^-binding loops. In the present case, the coordinating residues in EF-I and EF-III could be confirmed by paramagnetic titration experiments, where in Ca^2+^ is displaced by a trivalent Ce^3+^ in [Ca^2+^]_2_-EhCaBP6, which results in the broadening of the spectral signatures of the coordinating residues. The information thus derived was used in the final stages of structure determination and refinement. The upper bound distance constraints between the Ca^2+^ and residues at positions 1, 3, 5, 7, and 12 were thus varied from 3 Å to 4.5 Å to fix the Ca^2+^ within the two Ca^2+^-binding loops EF-I and EF-III.

All these constraints taken together (on an average 9 constraints per residue), five hundred structures were calculated, from which 20 structures with the lowest target function values were selected for structural analysis individually for both the N- and C-terminal domains (NTD and CTD) and also for the full length protein. The program PROCHECK-NMR available on PSVS-1.5 web server was used to estimate the quality of the lowest energy structures of NTD and CTD and validated them. The structures were visualized using VMD [56].The coordinates for full length [Ca^2+^]_2_-EhCaBP6 has been deposited in the PDB (pdb id: 5B7X). The corresponding structural statistics for NTD and CTD separately are given in Table 2.

### Culture of parasite

*E*. *histolytica* HM-1:IMSS strains were maintained and grown in TYI-S-33 medium containing 250 μg/ml benzyl penicillin and 0.25 mg/ml streptomycin per 100 ml of medium. The cells were then grown for 48 h (60–70% confluent) prior to carrying out experiments.

The transformed cells containing tetracycline inducible vector were grown in the presence of 10 μg/ml of Hygromycin B. For experiments, cells were grown for 48 h in the presence of 10 μg/ml Hygromycin B. The cells were serum starved for 24 h with incomplete media containing 0.5% of adult bovine serum. Post 24 h serum starvation; cells were replenished with complete media containing 15% adult bovine serum. 10 μg/ml of Hygromycin B as well as 30 μg/ml tetracycline was added and the cells were harvested at different time points (0–12 h).

### Transfection and selection of *E*. *histolytica* trophozoites

Electroporation was used for transfection of *E*. *histolytica*. In short, log phase culture was harvested and washed with 1X PBS pH 8.0. The harvested cells were washed twice with incomplete cytomix buffer (10 mM K_2_HPO_4_/KH_2_PO_4_ (pH 7.6), 120 mM KCl, 0.15 mM CaCl_2_, 25 mM HEPES (pH 7.4), 2 mM EGTA, 5 mM MgCl_2_). The cells were then resuspended in 0.8 ml complete cytomix buffer (incomplete cytomix buffer containing 4 mM ATP and 10 mM reduced Glutathione). A 100 μg of plasmid DNA was added to the cells and subjected to two consecutive pulses of 3,000 V cm^− 1^ (1.2 kV) at 25 mF (Bio-Rad, electroporator). The transfectants were initially allowed to grow without drug. However, after 48 h, 5 μg/ml Hygromycin B was added. Concentration of the drug was increased gradually to 10 μg/ml till a stable cell line was obtained.

### Cloning of EhCaBP6 sense and EhCaBP6 antisense

pEhHYG-tetR-O-CAT shuttle vector was used for cloning of sense and antisense constructs [57]. The CAT gene of pEhHYG-tetRO-CAT was excised using KpnI and BamHI and EhCaBP6 gene was inserted in its place in sense and antisense orientation. The oligonucleotides used for making the above stated constructs are shown below.

CaBP6 Sense Fp: 5’ CGG GGT ACC ATG TCT ATG GAA ATA GAA GC 3’

CaBP6 Sense Rp: 5’ GCG GGA TCC TTA AAG AGC AAC AAG TTT AA 3’

CaBP6 Antisense Fp: 5’ CGC GGA TCC ATG TCT ATG GAA ATA GAA GC 3’

CaBP6 Antisense Rp: 5’ GCC GGT ACC TTA AAG AGC AAC AAG TTT AA 3’

The constructs were then transfected as mentioned previously [57].

### Cloning of GFP-tagged fusion protein

The gene encoding EhCaBP6 was cloned in the BamH1 restriction site of pEh-Neo-GFP vector. The vector has been previously constructed by cloning the GFP mut3 allele of GFP [58] in the unique BamH1 site of the pExEhNeo plasmid [59]. Similarly, the gene for Calcium binding defective mutant was also cloned in the same vector at the C-terminus of GFP. The CAT gene of the shuttle vector pEhHYG-tetR-O-CAT was excised using KpnI and BamHI and replaced by the gene either in the sense or the antisense orientation.

### Cell synchronization and drug treatment

To obtain synchronous culture at G0/G1 phase, the cells were serum starved for 24 h with incomplete media containing 0.5% adult bovine serum. The cells were released from the G0/G1 arrest by transferring the culture to complete medium containing 15% adult bovine serum. In order to obtain cells at different phases, cells were harvested at different time point after serum replenishment. In case of transfectants, the cells were induced with 30 μg/ml of tetracycline after serum replenishment and were harvested at different time point.

### Immunofluorescence staining

Immunofluorescence staining was carried out as described previously [17]. *E*. *histolytica* cells, which were briefly resuspended in warm incomplete TYI-33 medium, were transferred onto acetone wiped cover slips placed in a 35 mm petri dish and allowed to adhere for 10 min at 37°C. The culture medium was removed and cells were fixed with 3.7% pre-warmed paraformaldehyde (PFA) for 30 min. After fixation, the cells were permeabilized with 0.1% Triton X-100/PBS for 3 min. The fixed cells were then washed with 1X PBS and neutralized for 30 min in 1X PBS containing 50 mM NH_4_Cl. The cover slips were blocked with 1% BSA/PBS for 1 h followed by incubation with primary antibody at 37°C for 1 h. The cover slips were washed with 1X PBS followed by 1% BSA/PBS before incubation with secondary antibody for 1 h at 37°C. Antibody dilutions were used as follows: Anti-m-EhCaBP6 at 1:300, anti-r-Eh β-tubulin at 1:50, anti-mice Alexa 555 at 1:300 and anti-rabbit Alexa 488 (Molecular Probes) at 1:300. The preparations were further washed with 1X PBS and stained with Hoechst (20 μg/ml) for 10 min at 37°C. The cells were washed thoroughly with 1X PBS and mounted on a glass slide using DABCO (1, 4-diazbicyclo [2,2,2] octane (Sigma) 10 mg/ml in 80% glycerol). The edges of the cover slips were sealed with nail-paint to avoid drying. For BAPTA-AM experiments, the cells were harvested and treated with 50, 100, 250, and 500 μM of BAPTA for 30 min prior to cell adherence in cover slips. The staining was further proceeded as mentioned above. The image analysis was done using NIS-Elements Analysis software (Nikon) that included merging of TRITC, FITC, DAPI and DIC channels, acquisition of intensity profile, determination of intensity at the region of interest and corresponding Pearson’s correlation co-efficient. For heat stress conditions, the cells were subjected to a temperature of 42°C for one hour.

### Immunoprecipitation from *E*. *histolytica* lysate

The HM1 total lysate (2 mg) was spun to remove cell debris and further pre-absorbed on Protein A-Sepharose beads (Preclearing). The purified anti-mice EhCaBP6 antibody was conjugated to CNBr-Sepharose beads in coupling buffer (0.1 M NaHCO_3_, 0.5 M NaCl, pH 8.6). The antibody coupled to CNBr-Sepharose beads was washed thrice with coupling buffer followed by blocking the unbound sites with 0.1 M Tris Glycine (pH 9.0). The antibody coupled CNBr beads were used to pull-down the antigen and the proteins binding to the antigen by incubating with the precleared lysate for an overnight at 4°C in a reaction volume of 200 μl. Immune complexes were centrifuged at 3000 g for 5 min followed by three washes each with buffer 1 (10 mM Tris-Cl (pH 7.5), 150 mM NaCl, 0.1% ovalbumin (w/v), 0.1% TritonX-100 (w/v), 0.05% sodium azide (w/v)) followed by buffer 2 (10 mM Tris-Cl pH 7.5, 150 mM NaCl) and buffer 3 (60 mM Tris-Cl (pH 6.8)). The pellet was resuspended in 50 μl of SDS PAGE buffer (125 mM Tris-Cl (pH 6.8), 2% SDS, 0.1% DTT, 30% glycerol, 5% β-mercaptoethanol, and bromophenol blue), and boiled for 5 min. The bound proteins were separated from beads by centrifugation for 5 min and the supernatant was analyzed by SDS-PAGE.

### Western analysis

For immunodetection, samples were separated on a 12% SDS–PAGE. The gel was then transferred on a polyvinylidine fluoride (PVDF) membrane and processed using standard methods. The antigens were detected with polyclonal antibodies raised in rabbit and mice followed by secondary anti-rabbit and anti-mice immunoglobulin conjugated to HRPO (1:10,000; Sigma). ECL reagents were used for visualization (Millipore). The concentration of protein in the sample was estimated by bicinchoninic acid (BCA) assay using BSA as a standard. SDS–PAGE analysis was carried out in 10–12% acrylamide gel under reducing conditions.

### Microtubule co-sedimentation assay

Co-sedimentation assay was carried out following the protocol from Tonglin Mao et. al [60]. The binding reaction was performed in 200 μl reaction volume containing 2 μM of bovine β-tubulin and 2 μM EhCaBP6 in polymerization buffer (1 mM MgCl_2_, 1 mM EGTA, and 100 mM PIPES-KOH (pH 6.9)) in the presence and absence of 1 mM GTP and 20 μM Taxol at 37°C for 1 h. After incubation, the samples were centrifuged at 100,000 g for 1 h at 37°C. The supernatant and pellet were separated and brought to equal volumes in SDS-PAGE buffer. The supernatant and pellet fraction were analyzed using 14% SDS-PAGE and visualized by staining gels with Coomassie Brilliant Blue R-250. EhCaBP6 alone and bovine β–tubulin alone were used as controls.

### Isothermal titration calorimetry

Isothermal titration calorimetry measurements were performed on a Microcal VP-ITC titration calorimeter at 25°C. Protein samples were decalcified, centrifuged and degassed prior to the titration with Ca^2+^. Each titration consisted of injecting 2/3 μL aliquots of 10 mM Ca^2+^ solution (diluted from 1 M standard CaCl_2_ solution supplied from Sigma-Aldrich chemicals) into 100–200 μM protein solution (1.7 mL) after every 3 minutes, to ensure that the titration peak returned to the baseline prior to the following injection. A total of 20 injections were carried out. Aliquots of concentrated ligand solutions were injected into the buffer solution (50 mM Tris-Cl (pH 7.0), 100 mM NaCl without the protein) in a separate ITC run, to subtract the heat of dilution. Three sets of titration were carried out in apo as well as holo form of the protein. The ITC data thus obtained was baseline corrected and analyzed using the software ORIGIN (*supplied with VP-ITC*). The amount of heat released per addition of the titrant was fitted to different models to find out the number of binding sites and the metal binding affinities of the protein. Repeating the experiment thrice with different concentrations of protein sample could reproduce the experimental data.

### Surface Plasmon Resonance

To characterize the interaction between Ehβ-tubulin and EhCaBP6, multiple sequence alignment of β–tubulin from human, *Dictyostelium*, bovine and *E*. *histolytica* was done (S13 Fig). The sequence alignment showed 56% sequence similarity among the organisms. Hence, we used commercially available Bovine β–tubulin for SPR studies. Bovine β–tubulin was resuspended in ice-cold 5% glycerol buffer (80 mM PIPES (pH 6.9), 2 mM MgCl_2_, 0.5 mM EGTA, 5% glycerol) and 10 μM was immobilized on a gold chip in 10 mM sodium acetate (pH 5.0) according to manufacturer’s amine coupling kit. After protein immobilization, the surface was blocked with 1 M ethanolamine (pH 8.5), followed by regeneration using 50 mM NaOH. The interaction experiments were performed using a buffer containing 10 mM HEPES (pH 7.4), 150 mM NaCl and 0.2 mM calcium chloride. We also performed interaction experiment in the absence of Ca^2+^ and supplementation with 5 mM of EGTA in the above buffer. The binding experiments were carried out with different concentrations (50, 100, 250, 500, 750, 1000 μM) of EhCaBP6 in running buffer and injected at a rate of 20 ml/min. The association kinetics was monitored for 300 s and dissociation kinetics was monitored for another 300 s. The data were recorded at 25°C and data analysis was performed using Biacore T2000 SPR Kinetics evaluation software.

### Cell proliferation assay

Growth kinetics was studied by inoculating equal number of cells (5000 cells/ml). The cells were allowed to grow at 35°C and harvested at 12, 24, 36, 48, and 72 h post inoculation. The cells were harvested in 1X PBS pH 7.2 by chilling on ice followed by centrifugation at 1500 g for 5 min. The cell pellet was resuspended in 1 ml 1X PBS pH 7.2. The cells were counted on Cell counter by mixing cells with 0.4% Trypan blue in a ratio of 1:1. At every time point, the cells were harvested and counted as mentioned above. Each experiment was performed in triplicate and the experiment was performed thrice. The standard error bars were calculated and represented.

### Thymidine incorporation assay

EhCaBP6 is a nuclear protein and it co-localizes with DNA during cell division in *E*. *histolytica*. Therefore, to study the effect of EhCaBP6 on rate of DNA synthesis, thymidine incorporation assay was done according to protocol mentioned in Nishant et al. [61]. Briefly, 7000 cells/ml were inoculated in complete media. Post inoculation cells were harvested at different time points (0, 12, 24, 36, 48, 72 h) and treated with ^3^H-Thymidine (10 μCi) for 2 h at 37°C. After incubation, cells were harvested and washed with ice chilled 1XPBS pH 8.0. To the cells 10% chilled TCA was added and incubated on ice for 45 min. The precipitate was collected on GFC glass microfiber filter using a vacuum pump. The filter was washed thrice each with 10% TCA followed by 5% TCA and final wash with 100% ethyl alcohol. The glass microfiber filter was dried at 37°C in a scintillation vial followed by 5 ml scintillation fluid before measurement using an imager.

### Cell viability assay

Cell viability assay was determined on the ability of cells to exclude the dye Trypan Blue. This assay was employed to assess the viability of cells during cell proliferation assay and H^3^-Thymidine incorporation assay. The cells harvested at given time point were mixed with 0.4% Trypan Blue in a ratio of 1:1 and mounted onto Countess cell counting chamber slide. The total number of cells along with number of live and dead cells were counted using program preset for *E*. *histolytica* trophozoites on the basis of cell size and shape in Countess automated cell counter (Invitrogen). The values obtained were plot in Microsoft Excel.

### FACS

To study if EhCaBP6 affected transition from G1 to S phase, flow cytometry was performed. The sense and the antisense cell lines of EhCaBP6 were synchronized at G0/G1 phase by 10 nM Hydroxyurea treatment for 12–14 h. In order to release the culture from G1 arrest, the hydroxyurea containing media was substituted with 12.5% serum supplemented media. The cells were then harvested at different time point to study transition from G0/G1 phase. The harvested cells were washed with ice-cold 1XPBS pH 7.2 and fixed using 3.7% paraformaldehyde. The fixed cells were washed thoroughly with chilled 1XPBS and treated with 0.3 mg/ml RNAaseA for 1 h at 37°C. The RNAase treated cells were stained with 0.04 mg/ml Propidium Iodide prepared in 0.6% TritonX-100 for 15 min in dark at 37°C. Flow cytometric analysis was carried out using FACSCalibur (Becton Dickinson, USA) equipped with a single-laser system (6 W Innova90-6 argon ion laser). For measurement of DNA content, cells were excited with 488 nm light and emission was measured through 575DF20 (for PI fluorescence; FL2). Data from 50,000 cells were recorded for each experiment and analyzed using Cell quest software (BD, USA).

### Western analysis

For immunodetection, samples were separated on 12% SDS–PAGE. The gel was then transferred on to a polyvinylidine fluoride membrane and processed using standard methods. The antigens were detected with polyclonal antibodies raised in rabbit and mice followed by secondary anti-rabbit and anti-mice immunoglobulins conjugated to HRPO (1:10,000, Sigma). ECL reagents were used for visualization (Millipore). The concentration of proteins in a sample was estimated by bicinchoninic acid assay using BSA as a standard. SDS–PAGE analysis was carried out in 10–12% acrylamide gels under reducing conditions according to the method of Laemmli.

### Microtubule polymerization assay

Bovine brain tubulin (Cytoskeleton) was resuspended in 200 μl of ice-cold General Tubulin Buffer (80 mM Pipes (pH 6.9), 2 mM MgCl_2,_ 0.5 mM EGTA and 1 mM GTP) for a final concentration of 5 mg/ml. The assays were performed on 96-well half-area plates with the concentration of EhCaBP6 varied (5, 10 and 50 μM), while keeping the concentration of tubulin constant at 5 μM. The reaction mixtures were prepared on half-area plates kept at 4°C and then transferred to a plate reader (Thermo Scientific MultiskanGo) preset at 37°C. The reading was collected in a kinetic mode by measuring the optical density every 30 s during 60 min at 340 nm. The data was then exported to Microsoft Excel and analyzed using GraphPad Prism 5 (GraphPad software, La Jolla, USA) software.

## Supporting information

S1 Fig (A) FPLC profile of EhCaBP6 and purified EhCaBP6 on SDS-PAGE (15%). (B) Matrix-assisted laser desorption ionization (MALDI) data of purified EhCaBP6. (C) Dynamic light scattering regularization plot of EhCaBP6 showing hydrodynamic radii (R_H_). (D) Far-UV CD spectra of EhCaBP6 at 25°C showing predominantly α–helical conformation.
(TIF)

S2 Fig 2D [^15^N-^1^H]-HSQC spectrum in going from (A) apo to (B) holo-EhCaBP6.
(TIF)

S3 Fig Ca^2+^-titration of apo- to holo-EhCaBP6 as monitored through the downfield shifted Gly signatures. The sequential occupancy was monitored by the sequential appearance of Gly peak corresponding to EF-I (G28) followed by EF-III (G101).
(TIF)

S4 Fig 2D-[^15^N-^1^H]-HSQC spectra of mutant (D23A, D25A, D96A and D98A) (A) In the presence of Ca^2+^ and (B) In the presence of EDTA. (C) An overlay of 2D-[^15^N-^1^H]-HSQC spectra of mutant in the presence of Ca^2+^ (blue) and in the presence of EDTA (red).
(TIF)

S5 Fig A representative NMR derived solution structure of full length [Ca^2+^]_2_-EhCaBP6 having the least residual target function value among the 20 conformers generated using CYANA. (TIF)

S6 Fig The Ca^2+^-binding loop of the unusual EF-hand I of [Ca^2+^]_2_-EhCaBP6 exhibiting octahedral Ca^2+^-coordination geometry. (TIF)

S7 Fig EhCaBP6 does not participate in erythrophagocytosis. Immunostaining of cells undergoing erythrophagocytosis with antibodies against EhCaBP1, EhCaBP3, EhCaBP5 and EhCaBP6 from *E*. *histolytica*. (A) EhCaBP1 and EhCaBP5 are enriched near phagocytic cups (marked by a *), EhCaBP3 is found during the closure of phagocytic cups and in mature phagosomes (marked by an arrow). EhCaBP6 was neither found near phagocytic cups nor in mature phagosomes. The secondary antibody used was anti-mice or rabbit- Alexa Fluor 488 (green) in a ratio of 1:300. (B) Densitometry analysis of EhCaBPs at phagocytic cup, in phagosome and nucleus plotted as a function of percentage relative intensity. A total of five regions of interest (ROI) were taken for analysis in each cell in the respective compartments. The sample size contained 50 cells per experiment. Each of these experiments was repeated three times.
(TIF)

S8 Fig EhCaBP6 binds Eh β-tubulin *in-vivo*. Antibody against EhCaBP6 was used to pull down EhCaBP6 and its binding partner Eh β-tubulin from whole-cell lysate of *E*. *histolytica* and proteins present in the precipitated material were identified by specific antibodies in western blots as indicated. Whole cell lysates were prepared in presence of either CaCl_2_ or EGTA. Prebleeds of indicated antibodies were used for immunostaining as control (lane I and IV). Lanes II and V represent immunoprecipitation in the presence of CaCl_2_ and lane III and VI show immunoprecipitation profile in the presence of EGTA. The total input lysate was also probed for the presence of EhCaBP6 and Eh β-tubulin by their respective antibodies (Lane VII and VIII). Anti-m-EhCaBP6 and anti-R- Eh β-tubulin were used at a dilution of 1:2000 and 1:300, respectively.
(TIF)

S9 Fig Surface Plasmon Resonance study of the interaction between monomeric β–tubulin and EhCaBP6. In the presence of (A) 1.5 mM of CaCl_2_ and (B) 5 mM of EGTA.
(TIF)

S10 Fig Depletion of intracellular Ca^2+^ leads to translocation of EhCaBP6 from nucleus to cytoplasm. (A) Immunostaining of EhCaBP6 in BAPTA-AM untreated and treated cells. EhCaBP6 (green) was probed with anti-m-EhCaBP6 and anti-m-Alexa-Flour 488 secondary antibody. The nucleus was stained with DAPI. (B) Densitometry analysis of EhCaBP6 in nucleus and cytosol. A total of five random regions of interest (ROI) were chosen from nucleus and cytosol from each cell and the intensity was determined. The sample size contained 50 cells per experiment. Each of these experiments was repeated three times. This panel shows the relative intensities (%) of EhCaBP6 present in nucleus and cytosol. (C) Subcellular fractionation of normal HM1 cells and cells treated with 500 μM BAPTA-AM. Total lysate from BAPTA-AM untreated and treated cells were fractionated into nuclear, cytosol and membrane fractions. The fractions were probed with anti-m-EhCaBP6. The blots were also probed with antibodies against Eh-fibrillarin (I), Eh-coactosine (II), and Eh-TMK9 (III) as markers for nuclear, cytosolic and membrane fractions, respectively.
(TIF)

S11 Fig Isothermal calorimetry. Thermogram of Ca^2+^-binding to the double negative mutant of EhCaBP6. The protein concentration was 145 μM in 50 mM Tris-HCl (pH = 7.0) containing 100 mM NaCl.
(TIF)

S12 Fig Nuclear localization of EhCaBP6 is an indirect effect of intracellular Ca^2+^ depletion (A) Expression analysis of GFP-native EhCaBP6 and GFP-DNM6 *in vivo* upon induction with varying G418 concentration. The total lysate was probed with anti-GFP antibody. *E*. *histolytica* trophozoites transfected with GFP vector alone was used as control. (B) Immunostaining of GFP constructs (GFP-EhCaBP6, GFP-DNM6, GFP-vector) in Paraformaldehyde fixed cells with anti-GFP antibody (1:300) and anti-EhCaBP6 antibody (1:300). The fluorescence conjugated secondary antibody (Alexa-488 (green), Alexa -555 (red)) were used to probe the primary antigen. (C) Quantitative analysis of the relative intensity in nucleus and cytoplasm using NIS-Elements Analysis software (Nikon) by taking into consideration 10 region of interest (ROI) in the nucleus and cytoplasm. The experiment was performed thrice. The representative data is an average of ROI from three independent experiments.
(TIF)

S13 Fig (A) Multiple sequence alignment of β–tubulin from Human, *Dictyostelium*, Bovine and *E*. *histolytica* done by ClustalW. (B) Percent identity matrix as determined by ClustalW.
(TIF)

S1 Table Quantitative analysis of cell population in percent obtained at different phases of one cell division cycle. (DOCX)
