# Supplementary figures and images for: Structure of Ca2+-binding protein-6 from Entamoeba histolytica and its involvement in trophozoite proliferation regulation
Source: PLoS Pathog. 2017 May 15;13(5):e1006332. doi: 10.1371/journal.ppat.1006332 (PMC5444848; doi:10.1371/journal.ppat.1006332)

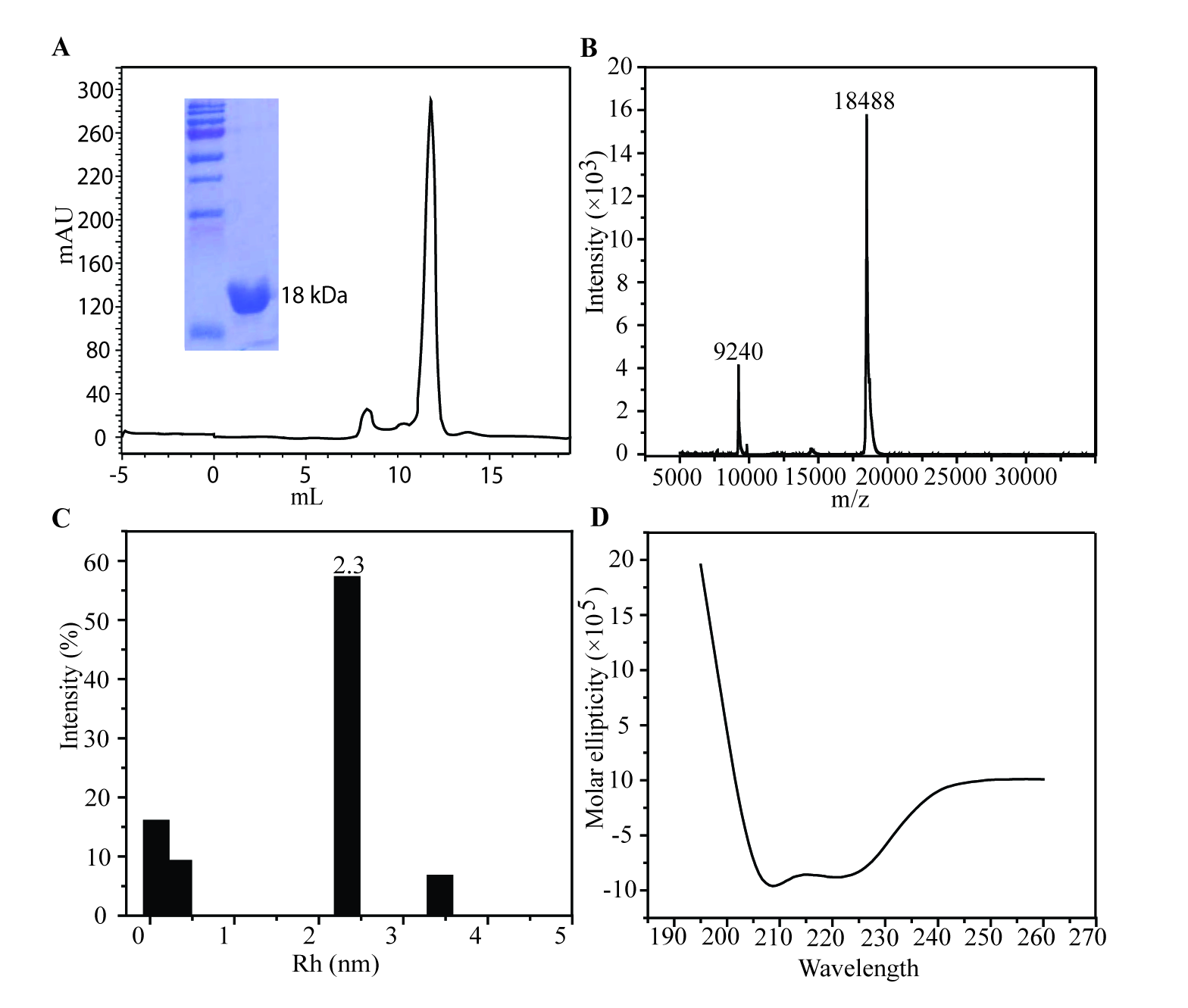

Supplement: S1 Fig — (A) FPLC profile of EhCaBP6 and purified EhCaBP6 on SDS-PAGE (15%). (B) Matrix-assisted laser desorption ionization (MALDI) data of purified EhCaBP6. (C) Dynamic light scattering regularization plot of EhCaBP6 showing hydrodynamic radii (RH). (D) Far-UV CD spectra of EhCaBP6 at 25°C showing predominantly α–helical conformation. (TIF) [file ppat.1006332.s001.tif]

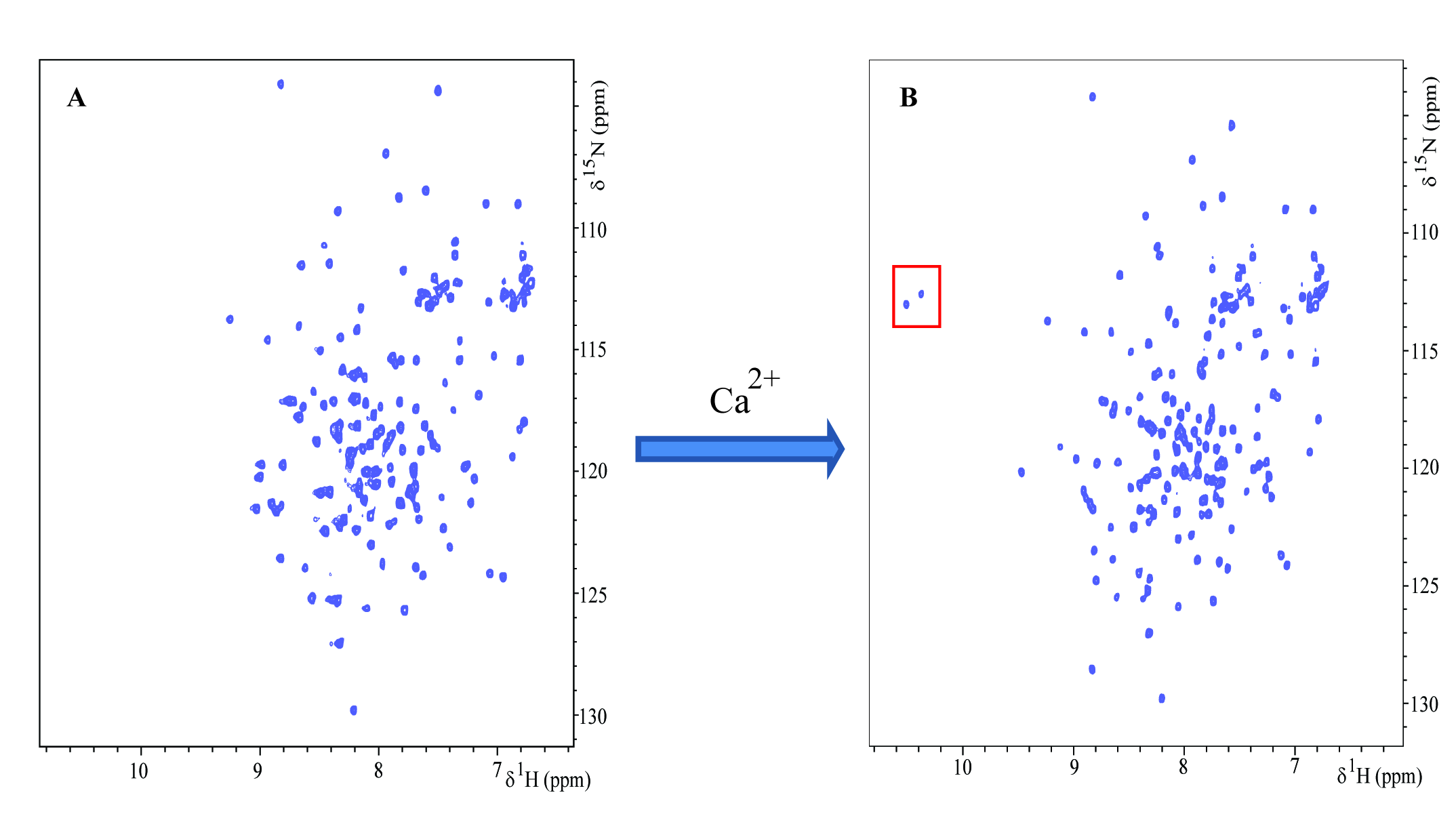

Supplement: S2 Fig — 2D [15N-1H]-HSQC spectrum in going from (A) apo to (B) holo-EhCaBP6. (TIF) [file ppat.1006332.s002.tif]

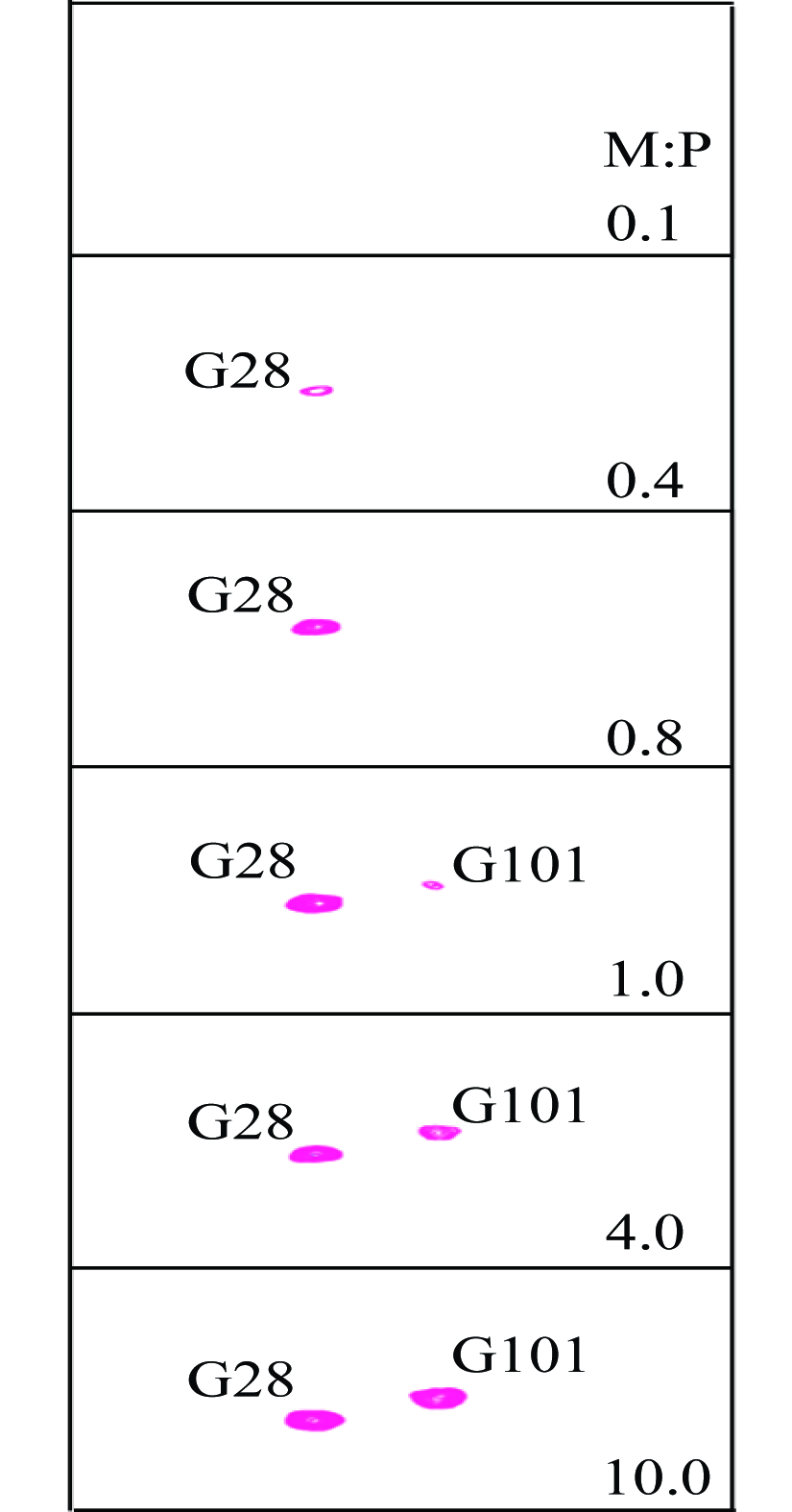

Supplement: S3 Fig — The sequential occupancy was monitored by the sequential appearance of Gly peak corresponding to EF-I (G28) followed by EF-III (G101). (TIF) [file ppat.1006332.s003.tif]

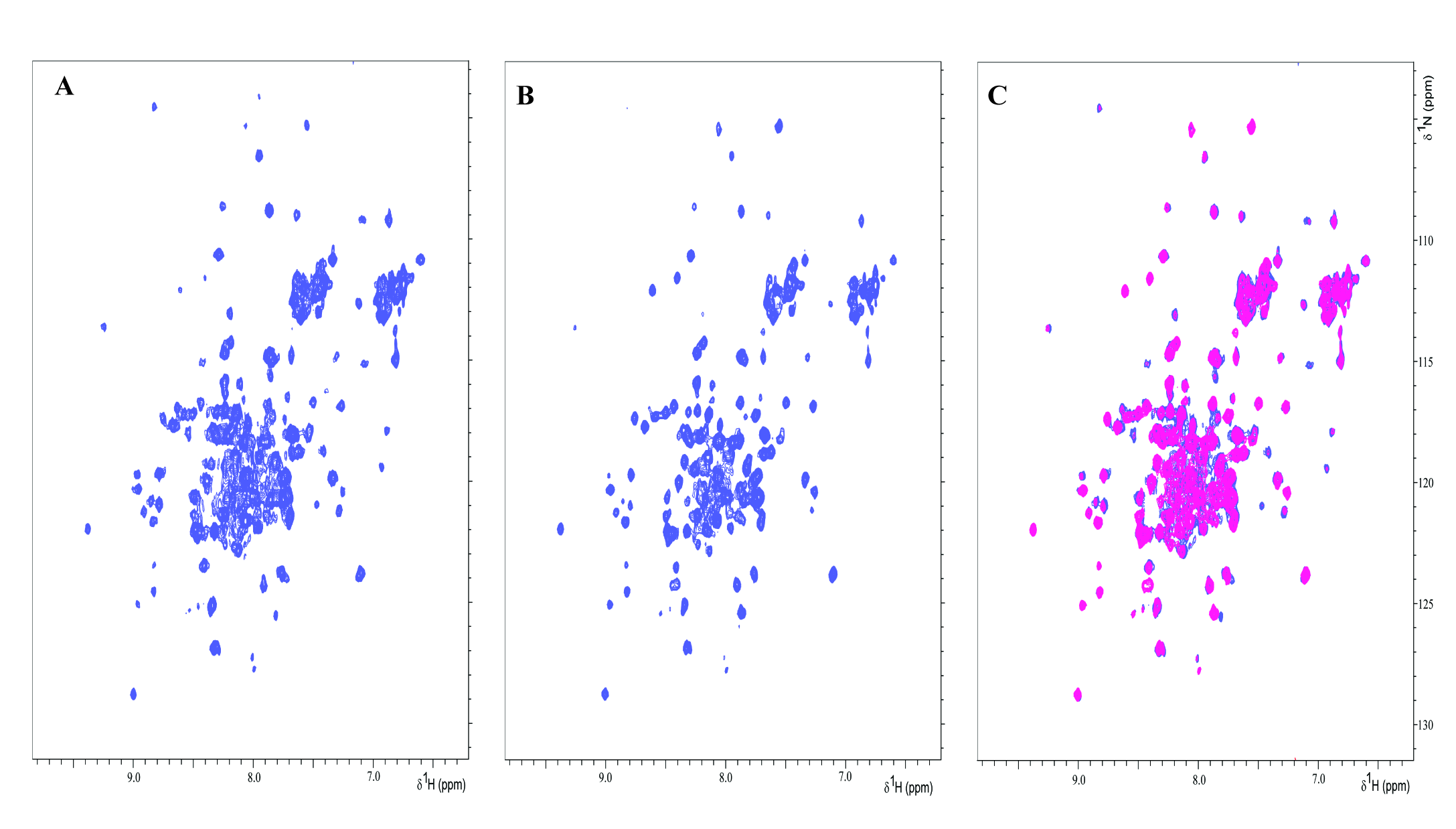

Supplement: S4 Fig — 2D-[15N-1H]-HSQC spectra of mutant (D23A, D25A, D96A and D98A) (A) In the presence of Ca2+ and (B) In the presence of EDTA. (C) An overlay of 2D-[15N-1H]-HSQC spectra of mutant in the presence of Ca2+ (blue) and in the presence of EDTA (red). (TIF) [file ppat.1006332.s004.tif]

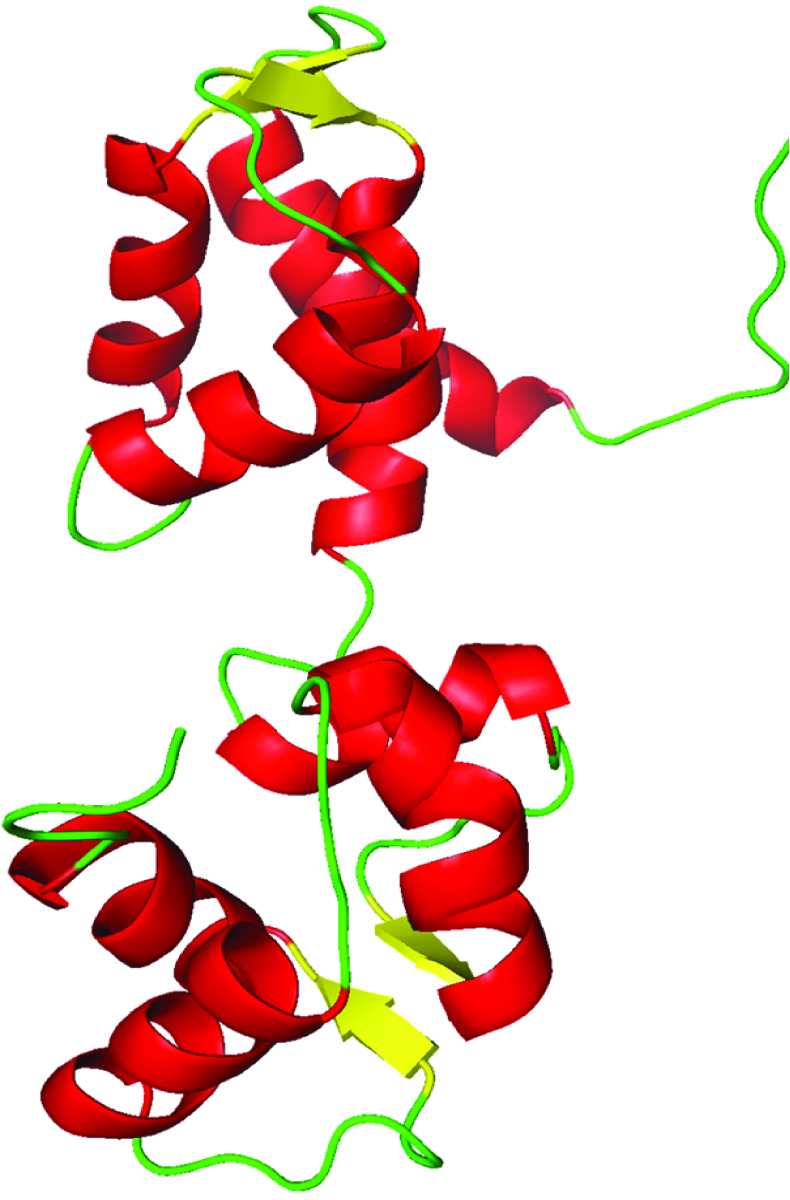

Supplement: S5 Fig — (TIF) [file ppat.1006332.s005.tif]

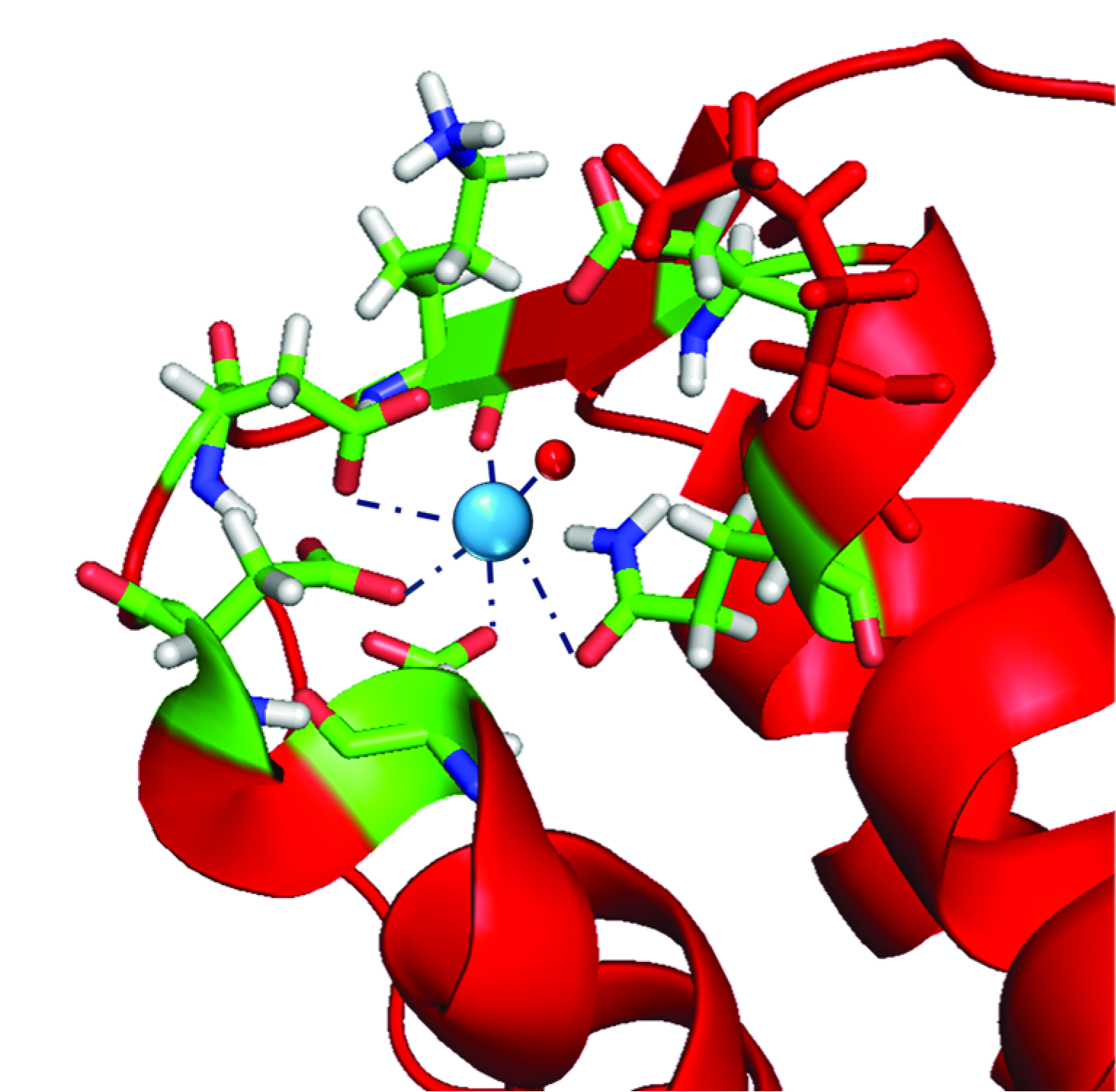

Supplement: S6 Fig — (TIF) [file ppat.1006332.s006.tif]

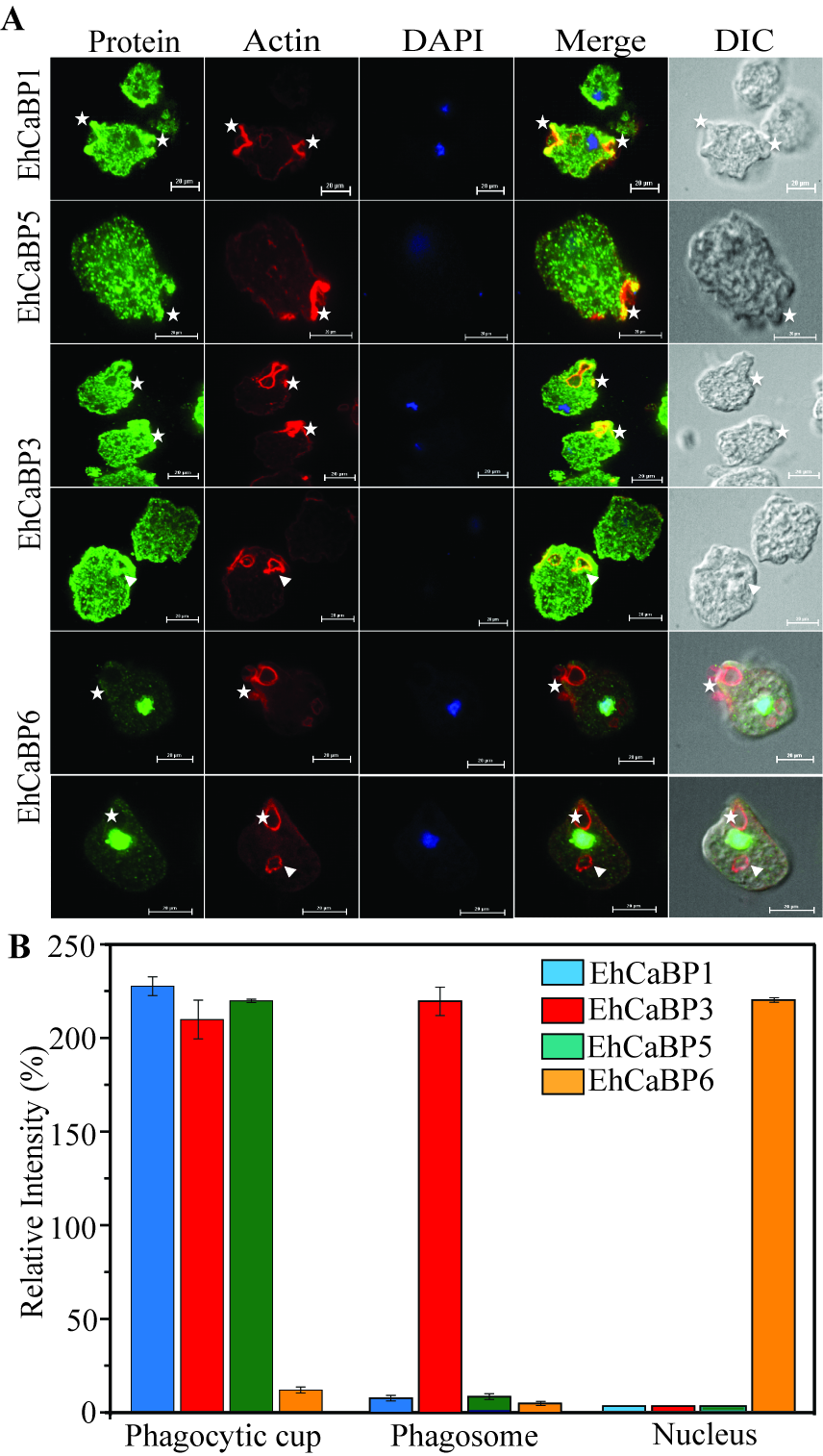

Supplement: S7 Fig — Immunostaining of cells undergoing erythrophagocytosis with antibodies against EhCaBP1, EhCaBP3, EhCaBP5 and EhCaBP6 from E. histolytica. (A) EhCaBP1 and EhCaBP5 are enriched near phagocytic cups (marked by a *), EhCaBP3 is found during the closure of phagocytic cups and in mature phagosomes (marked by an arrow). EhCaBP6 was neither found near phagocytic cups nor in mature phagosomes. The secondary antibody used was anti-mice or rabbit- Alexa Fluor 488 (green) in a ratio of 1:300. (B) Densitometry analysis of EhCaBPs at phagocytic cup, in phagosome and nucleus plotted as a function of percentage relative intensity. A total of five regions of interest (ROI) were taken for analysis in each cell in the respective compartments. The sample size contained 50 cells per experiment. Each of these experiments was repeated three times. (TIF) [file ppat.1006332.s007.tif]

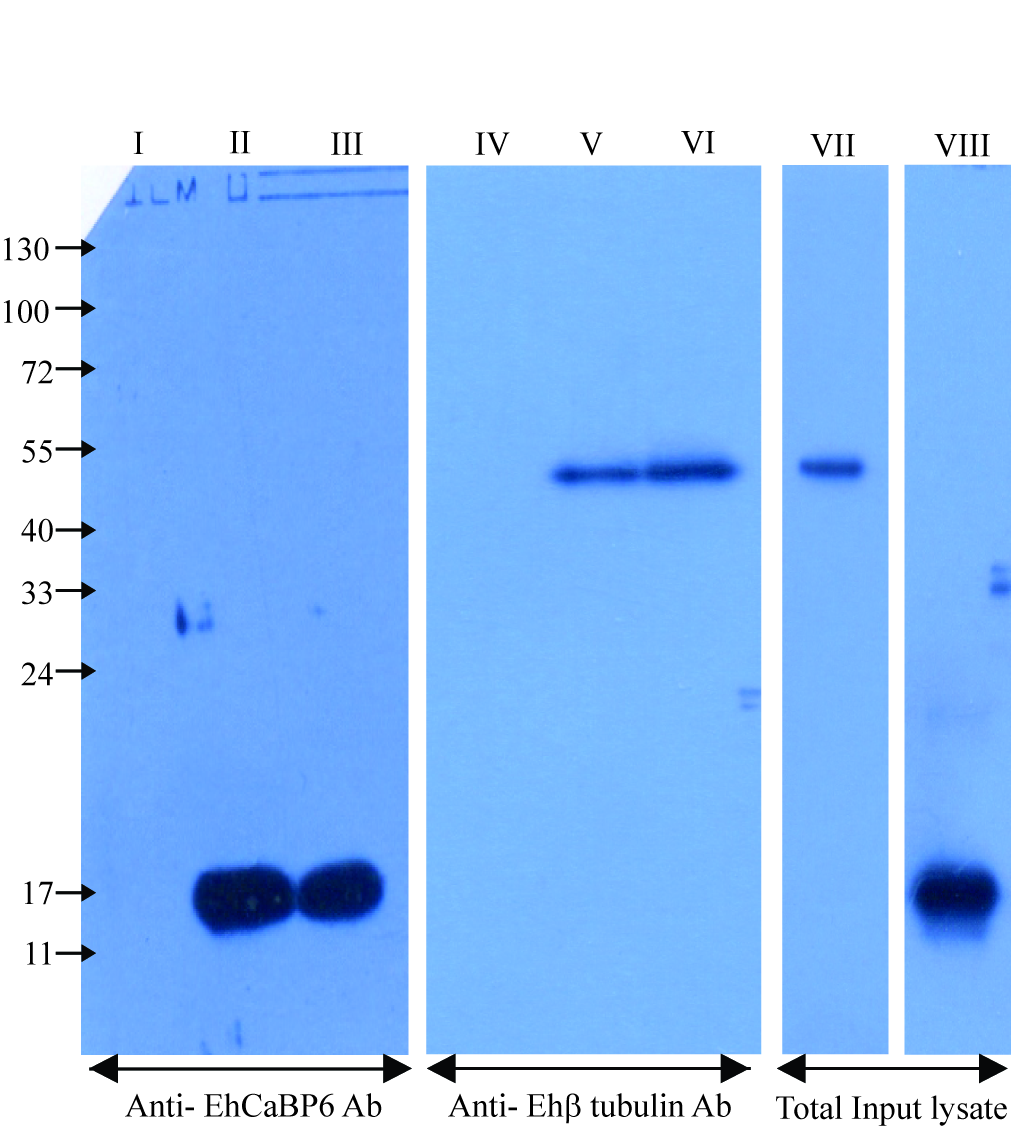

Supplement: S8 Fig — Antibody against EhCaBP6 was used to pull down EhCaBP6 and its binding partner Eh β-tubulin from whole-cell lysate of E. histolytica and proteins present in the precipitated material were identified by specific antibodies in western blots as indicated. Whole cell lysates were prepared in presence of either CaCl2 or EGTA. Prebleeds of indicated antibodies were used for immunostaining as control (lane I and IV). Lanes II and V represent immunoprecipitation in the presence of CaCl2 and lane III and VI show immunoprecipitation profile in the presence of EGTA. The total input lysate was also probed for the presence of EhCaBP6 and Eh β-tubulin by their respective antibodies (Lane VII and VIII). Anti-m-EhCaBP6 and anti-R- Eh β-tubulin were used at a dilution of 1:2000 and 1:300, respectively. (TIF) [file ppat.1006332.s008.tif]

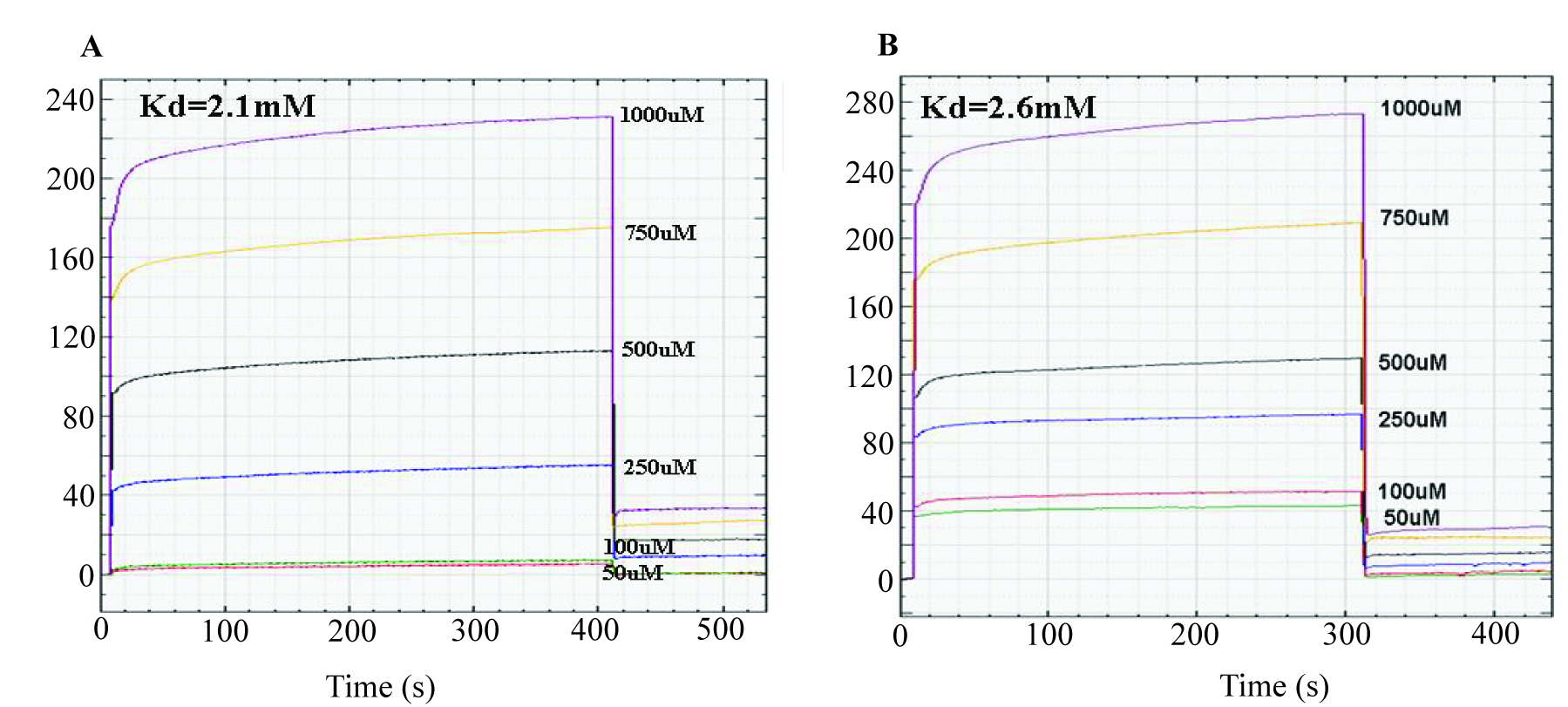

Supplement: S9 Fig — In the presence of (A) 1.5 mM of CaCl2 and (B) 5 mM of EGTA. (TIF) [file ppat.1006332.s009.tif]

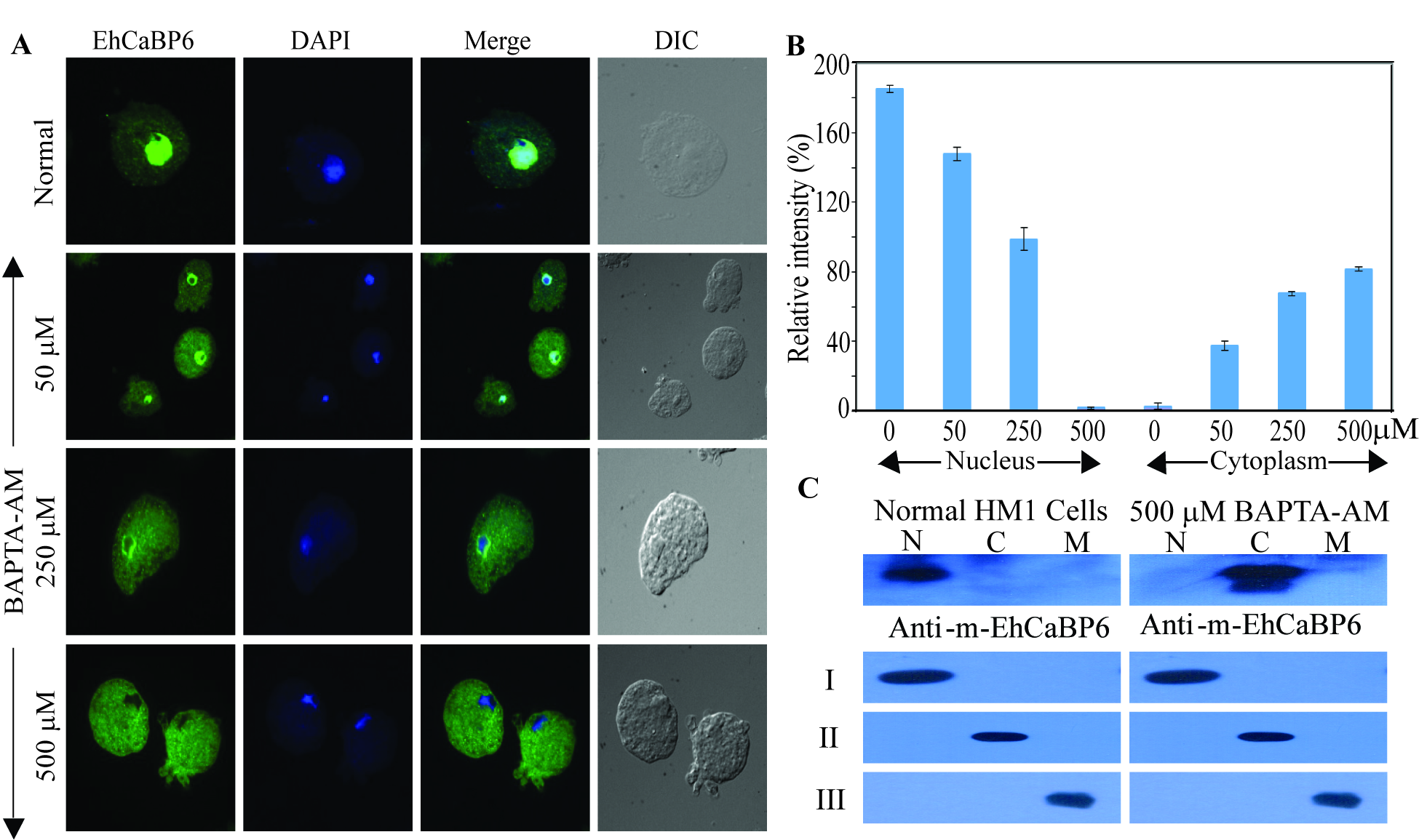

Supplement: S10 Fig — (A) Immunostaining of EhCaBP6 in BAPTA-AM untreated and treated cells. EhCaBP6 (green) was probed with anti-m-EhCaBP6 and anti-m-Alexa-Flour 488 secondary antibody. The nucleus was stained with DAPI. (B) Densitometry analysis of EhCaBP6 in nucleus and cytosol. A total of five random regions of interest (ROI) were chosen from nucleus and cytosol from each cell and the intensity was determined. The sample size contained 50 cells per experiment. Each of these experiments was repeated three times. This panel shows the relative intensities (%) of EhCaBP6 present in nucleus and cytosol. (C) Subcellular fractionation of normal HM1 cells and cells treated with 500 μM BAPTA-AM. Total lysate from BAPTA-AM untreated and treated cells were fractionated into nuclear, cytosol and membrane fractions. The fractions were probed with anti-m-EhCaBP6. The blots were also probed with antibodies against Eh-fibrillarin (I), Eh-coactosine (II), and Eh-TMK9 (III) as markers for nuclear, cytosolic and membrane fractions, respectively. (TIF) [file ppat.1006332.s010.tif]

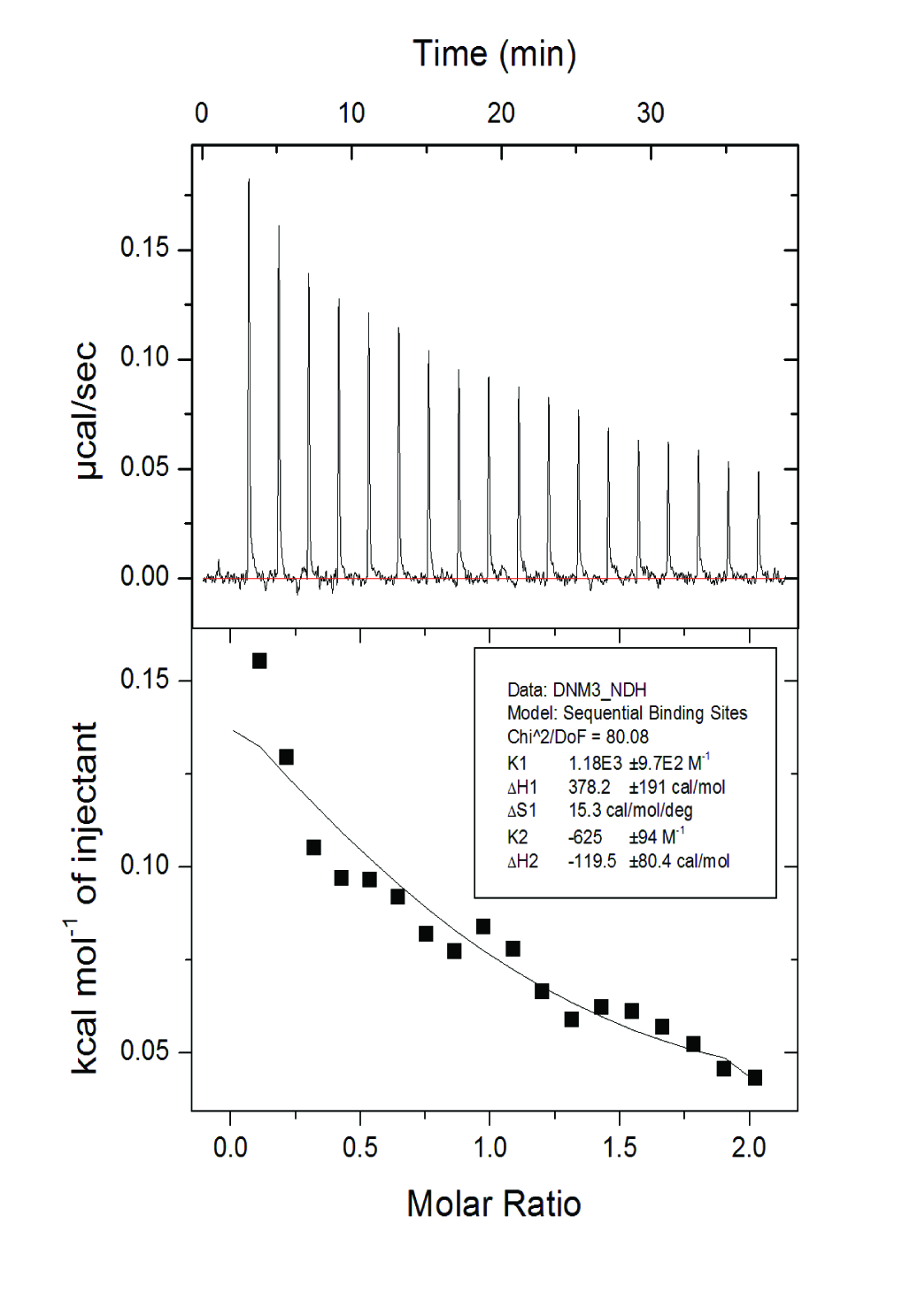

Supplement: S11 Fig — Thermogram of Ca2+-binding to the double negative mutant of EhCaBP6. The protein concentration was 145 μM in 50 mM Tris-HCl (pH = 7.0) containing 100 mM NaCl. (TIF) [file ppat.1006332.s011.tif]

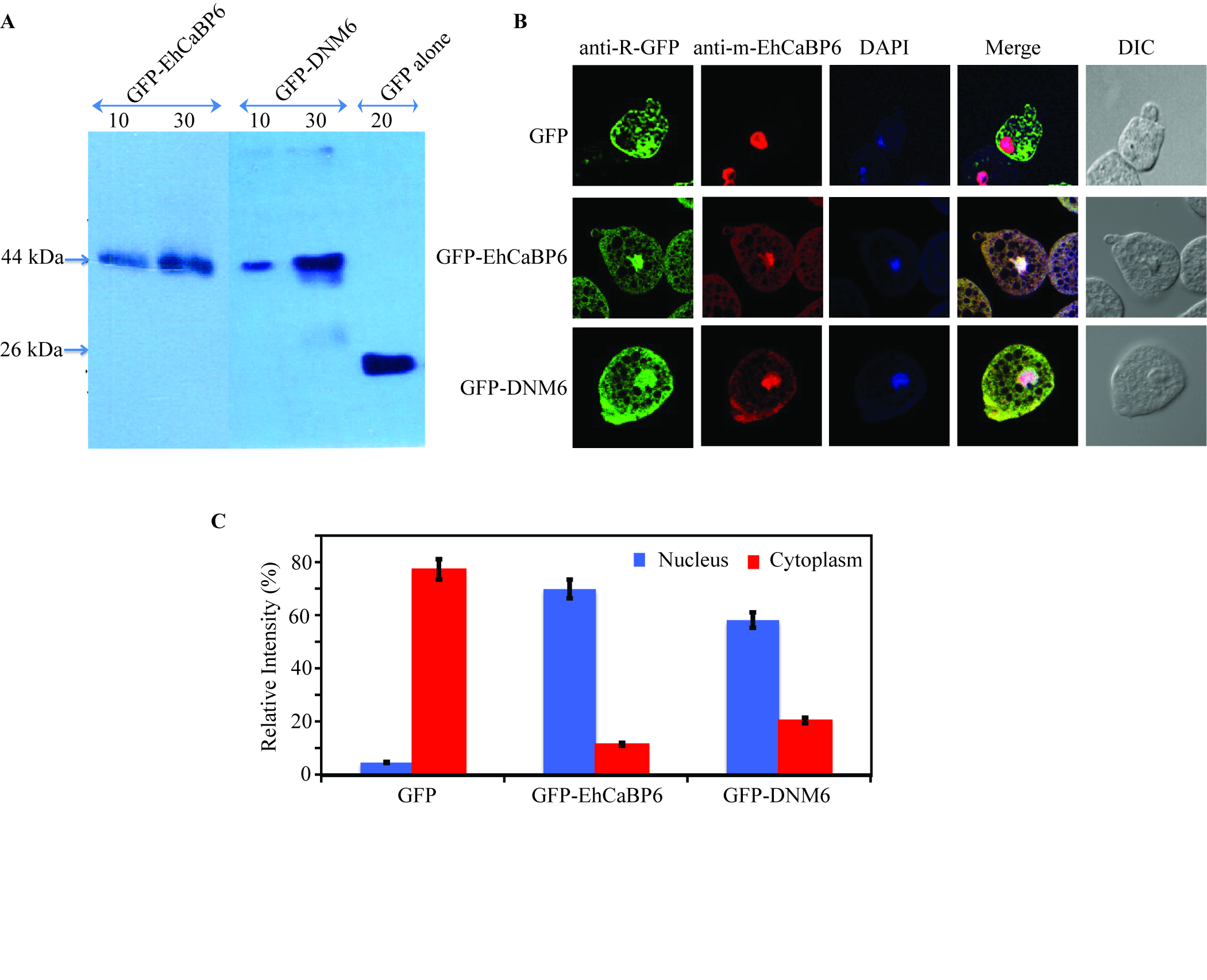

Supplement: S12 Fig — (A) Expression analysis of GFP-native EhCaBP6 and GFP-DNM6 in vivo upon induction with varying G418 concentration. The total lysate was probed with anti-GFP antibody. E. histolytica trophozoites transfected with GFP vector alone was used as control. (B) Immunostaining of GFP constructs (GFP-EhCaBP6, GFP-DNM6, GFP-vector) in Paraformaldehyde fixed cells with anti-GFP antibody (1:300) and anti-EhCaBP6 antibody (1:300). The fluorescence conjugated secondary antibody (Alexa-488 (green), Alexa -555 (red)) were used to probe the primary antigen. (C) Quantitative analysis of the relative intensity in nucleus and cytoplasm using NIS-Elements Analysis software (Nikon) by taking into consideration 10 region of interest (ROI) in the nucleus and cytoplasm. The experiment was performed thrice. The representative data is an average of ROI from three independent experiments. (TIF) [file ppat.1006332.s012.tif]

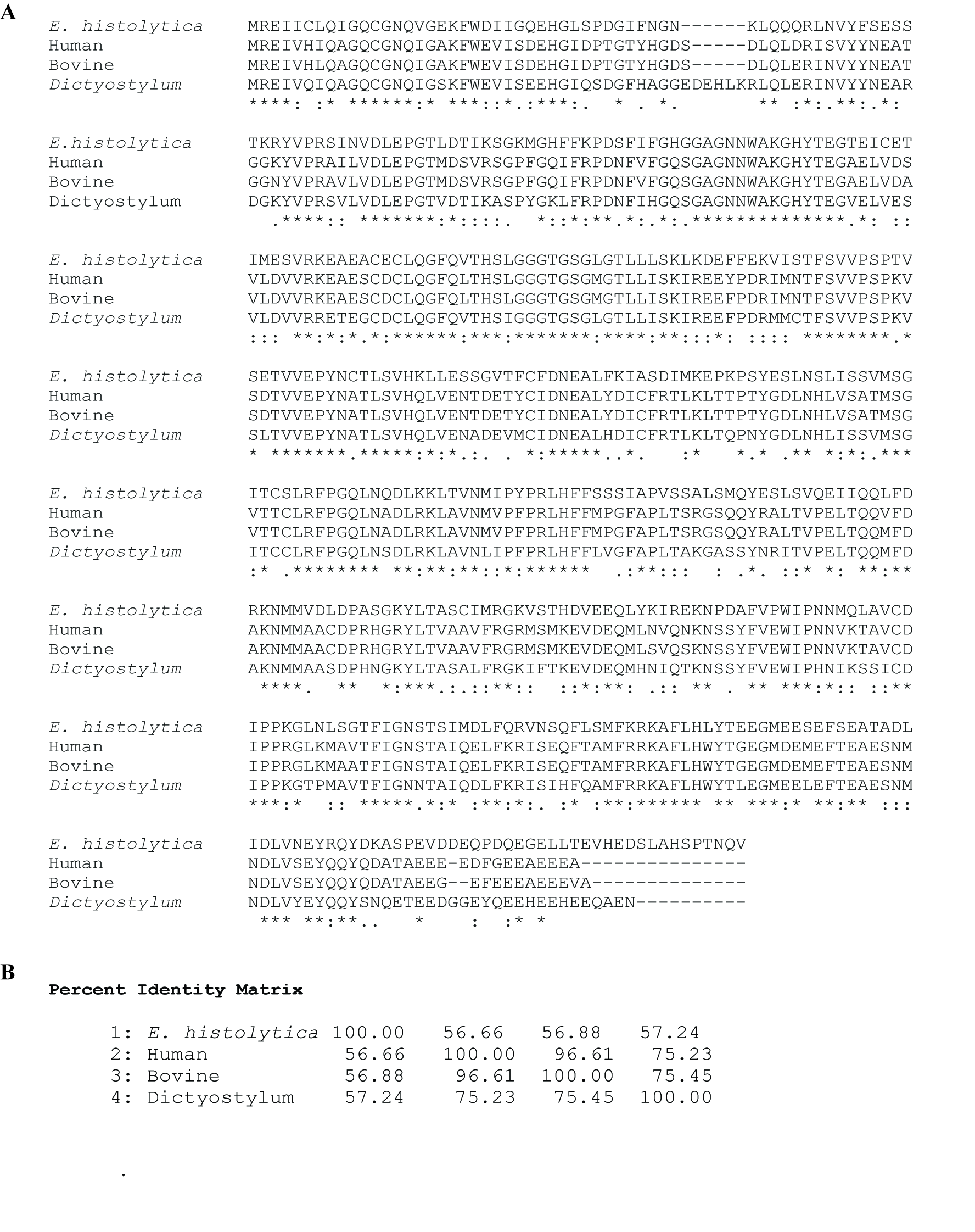

Supplement: S13 Fig — (A) Multiple sequence alignment of β–tubulin from Human, Dictyostelium, Bovine and E. histolytica done by ClustalW. (B) Percent identity matrix as determined by ClustalW. (TIF) [file ppat.1006332.s013.tif]
