# Supplementary material for: Structure of Ca2+-binding protein-6 from Entamoeba histolytica and its involvement in trophozoite proliferation regulation
Source: PLoS Pathog. 2017 May 15;13(5):e1006332. doi: 10.1371/journal.ppat.1006332 (PMC5444848; doi:10.1371/journal.ppat.1006332)
Supplement: S1 Table — (DOCX) [file ppat.1006332.s014.docx]

|  | 0 h | 4 h | 6 h | 8 h | 10 h |
| --- | --- | --- | --- | --- | --- |
| Phase |  |  |  |  |  |
|  | **TOC (+tet)** | | | | |
| G1 | 99.01 | 79.60 | 22.80 | 24.42 | 69.36 |
| S | 0.99 | 20.44 | 77.20 | 73.73 | 0 |
| G2 | 0 | 0 | 0 | 1.80 | 30.64 |
|  | **Sense (+tet)** | | | | |
| G1 | 98.17 | 77.37 | 8.50 | 0 | 61.74 |
| S | 1.83 | 22.61 | 91.08 | 30.29 | 19.62 |
| G2 | 0 | 0 | 0 | 69.71 | 18.64 |
|  | **Anti-Sense (+tet)** | | | | |
| G1 | 91.08 | 86.16 | 84.24 | 66.73 | 75.01 |
| S | 8.33 | 14.84 | 16.76 | 33.27 | 24.99 |
| G2 | 0.59 | 0 | 0 | 0 | 0 |

**Table S1**. Percentage cell population during different phases of one cell division cycle.
